# Solitary Median Maxillary Central Incisor Syndrome: An Exploration of the Pathogenic Mechanism

**DOI:** 10.3389/fgene.2022.780930

**Published:** 2022-01-24

**Authors:** Jie Li, Dandan Liu, Yang Liu, Chenying Zhang, Shuguo Zheng

**Affiliations:** Department of Preventive Dentistry, Peking University School and Hospital of Stomatology and National Center of Stomatology and National Clinical Research Center for Oral Diseases and National Engineering Research Center of Oral Biomaterials and Digital Medical Devices, Beijing, China

**Keywords:** solitary median maxillary central incisor syndrome, SMMCI, 18p deletion, SNP array, whole-genome sequencing

## Abstract

This study aimed to identify the genetic cause of one Chinese family with solitary median maxillary central incisor (SMMCI) and explore the relationship between genotype and its phenotype. One Chinese family with clinical diagnosis of SMMCI was collected. Single Nucleotide Polymorphism (SNP) array was performed and identified variation was confirmed by whole-genome sequencing (WGS). The reported chromosomal abnormalities and pathogenic genes in patients with SMMCI in literature were reviewed and summarized. The proband was an 8-year-old boy presenting a typical solitary median maxillary central incisor with a range of other phenotypic anomalies, including ptosis. SNP array revealed a 14.3 Mbp heterozygous deletion at chromosome 18p11.32-p11.21 in the proband but not in the unaffected parents. WGS further confirmed the identified deletion. 194 genes were involved in the chromosome region. Among them, 12 genes had been shown to be associated with diseases, including *TGIF1*, a reported SMMCI gene. The *de novo* 18p deletion resulted in SMMCI in the present study. Our results provide new genetic evidence that structural abnormality in chromosome 18p contributes to solitary median maxillary central incisor.

## Introduction

Solitary median maxillary central incisor (SMMCI; MIM 147250) syndrome is a complex disorder consisting of multiple, developmental defects involving midline structures of the head, which includes the cranial bones, the maxilla, and related dentition (specifically the central incisor tooth germ), together with other midline structures of the body (Hall, 2006). It is an autosomal dominant genetic disease. The estimated incidence of SMMCI syndrome is 1:50,000 live births (Poelmans et al., 2015). Although it can be an isolated trait, the presence of a SMMCI can be part of a syndrome and predicts associated anomalies, particularly the holoprosencephaly (HPE) spectrum (Nanni et al., 2001), the inheritance pattern of which is also autosomal dominant.

Abnormalities in other midline structures of the body are common in SMMCI syndrome besides the SMMCI tooth. Congenital nasal malformation, including choanal atresia, midnasal stenosis, or congenital pyriform aperture stenosis, is frequently reported in SMMCI cases. Other Common congenital anomalies associated with SMMCI are the following: severe to mild intellectual disability, congenital heart disease, and cleft lip and/or palate. In addition, short stature is present in half of the patients (Nanni et al., 2001; Hall, 2006).

SMMCI has been described mainly as part of the spectrum of HPE, in which the brain does not separate into distinct hemispheres and is associated with neurologic impairment and dysmorphism of the brain and face (Fallet-Bianco, 2018; Monteagudo, 2020). The most severe cases of HPE are not compatible with life and often appear as spontaneous abortions, while the less severe cases can be characterized by a SMMCI. Although they are generally mildly affected, patients with SMMCI belong to the HPE spectrum and are at risk to have children with more severe forms of HPE (Marini et al., 2003; El-Jaick et al., 2007). SMMCI has also been sporadically described in other non-HPE conditions, some well recognized, such as CHARGE syndrome, VACTERL syndrome, velocardiofacial (VCF) syndrome, ectodermal dysplasia, and DiGeorge syndrome (Hall, 2006).

Here, our group reported a sporadic SMMCI case. A 14.3 Mbp heterozygous deletion at chromosome 18p11.32-p11.21 was identified by Single Nucleotide Polymorphism (SNP) array in the proband. Whole-genome sequencing (WGS) further confirmed the identified deletion and found 12 genes that had been shown to be associated with diseases in the chromosome region, including *TGIF1*, a reported SMMCI gene.

## Materials and Methods

### Participants

This study was ethically approved by the Ethical Committee of Peking University School and Hospital of Stomatology (issue number: PKUSSIRB-201840184) and was conducted following the World Medical Association’s Declaration of Helsinki. All participants or their guardians signed written informed consent.

The proband was a boy with the age of 8 years old, who was referred to Peking university school and hospital of stomatology with the main complaint of only one maxillary central incisor eruption. Detailed dental treatment history and past medical history were recorded. Clinical and radiographic examinations were performed, and he was diagnosed as SMMCI according to the criteria of clinical diagnosis of SMMCI (Hall, 2006). Detailed clinical examinations were also performed for the proband’s family members.

### Mutation Analysis

Peripheral blood samples were collected from the participants and genomic DNA was extracted using TIANamp Blood DNA mini kit (Tiangen, Beijing, China) following the manufacturer’s instruction. The exons and exon-intron boundaries of the *SHH* gene were amplified by polymerase chain reaction (PCR) using the intron-exon specific primers as described previously (El-Jaick et al., 2007). In brief, the PCR reactions were carried out in a DNA Engine PTC-200 (Bio-Rad Laboratories, Hercules, CA, United States) using the program described elsewhere (Zhang et al., 2017). The amplification products were assessed by 1.2% agarose gel electrophoresis. Purified PCR products were bi-directionally sequenced using an ABI 3730 XL automatic sequencer (Applied Biosystems, Foster City, CA). DNA sequences were analysed using the databases of NCBI and the BLASTN program (BLASTN, RRID:SCR_001598).

### SNP Array Analysis

SNP array was performed to detect the genomic variation on the Infinium Global Screening Array (Illumina, San Diego, CA, United States) following the manufacturer’s protocol. The SNP array experiments were performed by FindRare Medical Technology Co., LTD. (Beijing, China). In brief, genomic DNA was hybridized to the array, which includes 700,000 markers genome-wide tagging SNPs and markers targeting all regions of known cytogenetic importance. The array was scanned with the Illumina iScan system (Illumina iScan System, RRID:SCR_020128) (Illumina, San Diego, CA, United States). Molecular karyotype analysis was performed by GenomeStudio V2011.1 software (GenomeStudio, RRID:SCR_010973) (Illumina, San Diego, CA, United States). Raw data were uploaded in KaryoStudio software (Illumina, San Diego, CA, United States) and B allele frequency and log R ratio were calculated by normalization to a reference “cluster,” which was generated from a set of 150–300 clinical samples. B allele frequency = number of B alleles/(number of A+ B alleles). Under normal conditions, the blue dot is near 0 (representing AA), 0.5 (representing AB) and 1 (representing BB). Log R ratio = log2 (copy number of target fragment/copy number of the sample as a whole [generally 2]). Automated detection of copy number changes was carried out using the cnvPartition algorithm (versions 1.2.1 to 3.1.6) (CNVPartition, RRID:SCR_010925) in KaryoStudio software (Illumina, San Diego, CA, United States). All identified abnormalities were further characterized by visual inspection of the Log R and B allele frequency chromosomal plots. Chromosomal deletion segments greater than 100 kb in size and chromosomal duplication segments greater than 200 kb in size were reported. The results were interpreted by referring to the DECIPHER (DECIPHER, RRID:SCR_006552), Database of Genomic Variants (Database of Genomic Variants, RRID:SCR_007000), OMIM (OMIM, RRID:SCR_006437) databases, and literature screening.

### WGS and Bioinformatics

To further characterize the chromosome variation, low coverage WGS was carried out using genomic DNA from the proband. The WGS was performed by FindRare Medical Technology Co., LTD. (Beijing, China). Briefly, 2 μg genomic DNA was randomly broken into fragments of approximately 300 bp by Covaris. These fragments were end-repaired and A-tailed, followed by the ligation to oligonucleotide adapters to prepare DNA libraries. Next, the qualified DNA was sequenced using the Illumina HiSeq Xten PE150 platform (Illumina HiSeq X Ten, RRID:SCR_016385) (Illumina, San Diego, CA, United States) using the paired-end sequencing approach.

High-quality paired-end reads were aligned to UCSC h19 human reference genome using Burrows-Wheeler Aligner (BWA, RRID:SCR_010910) (Li and Durbin, 2009) with the default parameters. Then SNP and indel detection was performed using the GATK (GATK, RRID:SCR_001876) (McKenna et al., 2010), copy number variations (CNV) were identified using the CNVnator (CNVnator, RRID:SCR_010821) (Abyzov et al., 2011), and structural variants (SV) were detected using the CREST (CREST, RRID:SCR_005257) (Chen et al., 2016), followed by variations annotation using the Annovar (Wang et al., 2010). Copy Number (log2 ratio) was calculated by normalization to a reference, which is the whole-genome sequencing results of internal normal peripheral blood sample (1600174H). Copy Number (log2 ratio) = log2 (copy number of target fragment)–1. When Copy Number (log2 ratio) is equal to 0, which represents the normal condition (Copy Number = 2). When Copy Number (log2 ratio) is equal to −1, which represents the heterozygous deletion (Copy Number = 1). When Copy Number (log2 ratio) is less than or equal to −2, which represents the homozygous deletion (Copy Number = 0). Furthermore, screening and filtration of the variants were performed based on the proband’s clinical phenotypes. Variants’ pathogenicity was assessed according to Standards and guidelines for the interpretation of sequence variants published by the American College of Medical Genetics and Genomics (ACMG) in 2015. The variants were named according to the HGVS nomenclature.

## Results

### Clinical Findings of the SMMCI Patient

The proband was an 8-year-old boy presenting a typical SMMCI phenotype from a non-consanguineous family. The weight and height of the patient was 129 cm (25th centile) and 30 kg (50th–75th centile) respectively. Previous dental treatment history showed that there was no dental traumatic history and dental extraction history of the maxillary anterior teeth. The proband’s parents were not consanguineous. There were no unexpected serious adverse events and no drug-related adverse events throughout the pregnancy. The family members’ medical history revealed no significant systematic disease, allergy, or use of medication. Following a detailed examination by a dentist, SMMCI syndrome was diagnosed.

**FIGURE 1:**
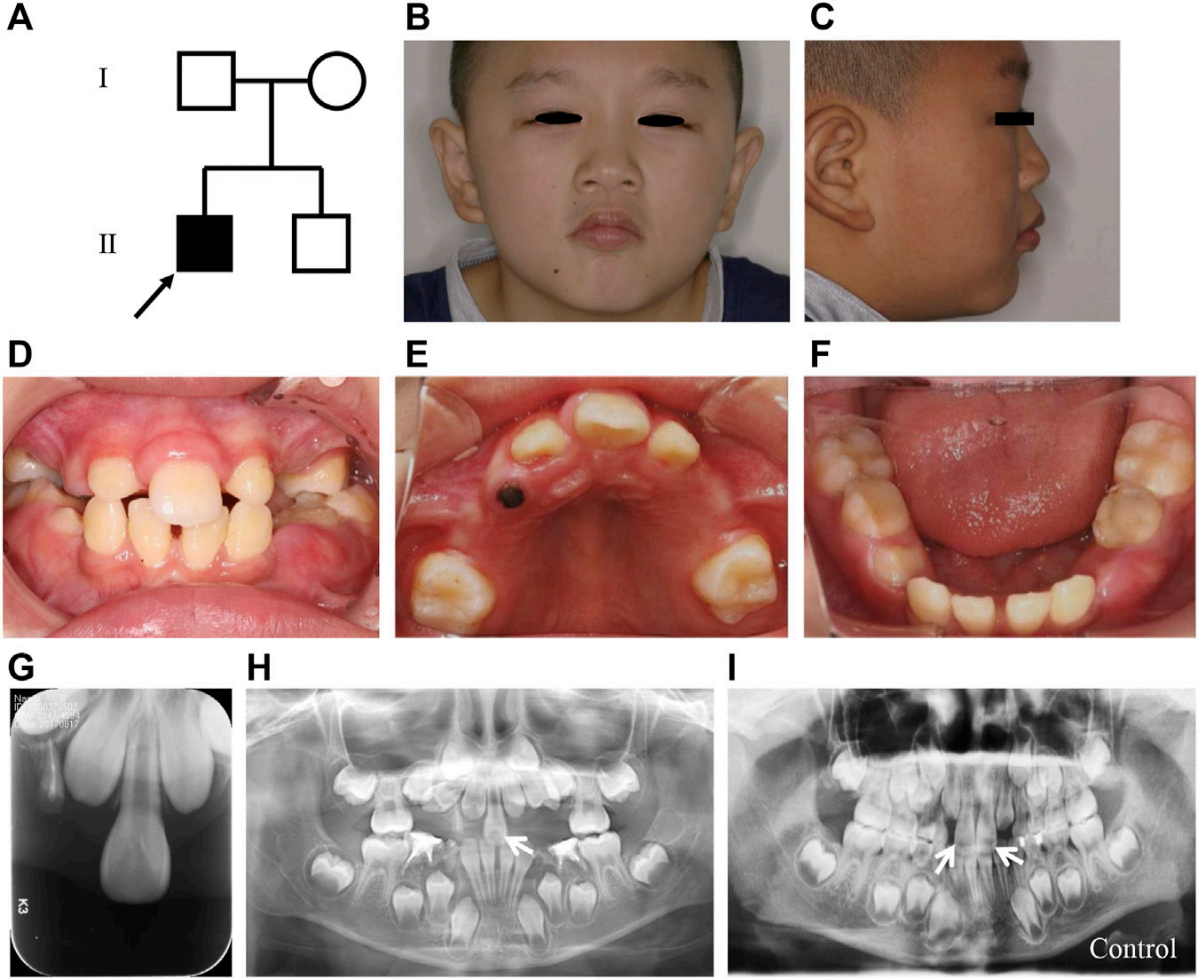
Clinical characteristics of the SMMCI patient in the present study. **(A)** Pedigree of the SMMCI patient. Males are represented by squares and females by circles. Filled symbols indicate affected individuals, and open symbols indicate those not affected. The arrow indicates the proband. **(B,C)** Frontal view **(B)** and lateral view **(C)** of the proband. **(D–F)** Intraoral photographs of the proband. **(G)** Periapical film of the proband. **(H)** Panoramic radiograph of the proband, the white arrow indicates the SMMCI tooth. **(I)** Panoramic radiograph of the normal control. White arrows indicate the normal maxillary teeth.

The pedigree of the patient contained four family members without similar manifestation (Figure 1A), indicating a sporadic feature of the case. Tonsillectomy and nasal polypectomy were performed when the patient was 6 years old. In addition, at 7 years old, surgery for correction of eyelid ptosis was performed. However, the feature of mild ptosis was still present after surgery (Figure 1B). The extra-oral photographs revealed that the proband had a characteristic, arch-shaped upper lip and an indistinct philtrum (Figures 1B,C). Besides, protruding ears were also shown (Figures 1B,C).

Oral examination showed that the patient was in mixed dentition, with only one maxillary central incisor erupted and located in the middle of the midline (Figures 1D,E), which was quite different from the normal two mandible control incisors (Figure 1F). Notably, the crown size of the erupted maxillary central incisor was similar to that of the normal central incisor and was symmetrical (Figure 1D). In addition, many primary teeth were premature loss, including primary maxillary and mandible canines and molars. Furthermore, the patient lacked the fraenulum of the upper lip and incisive papilla (Figures 1D,E). Narrowing upper dental arch and high vault were also shown in the patient and the midpalatal ridge was prominent (Figure 1E).

The periapical film and panoramic radiography showed that there was only one central incisor in the maxillary (Figures 1G,H) comparing to the two maxillary central incisors from a healthy age- and gender-matched child (Figure 1I). In addition, impacted teeth, supernumerary teeth, and undeveloped remaining tooth germs were all absent in the patient.

### Molecular Analysis

To explore the pathogenic mechanism of the sporadic case, mutation analysis of the *SHH* gene was first performed, which was the most reported gene been associated with SMMCI. The coding region and adjacent intron boundaries of the *SHH* gene were amplified by PCR followed by direct sequencing. However, no mutation was detected (data not shown).

**FIGURE 2:**
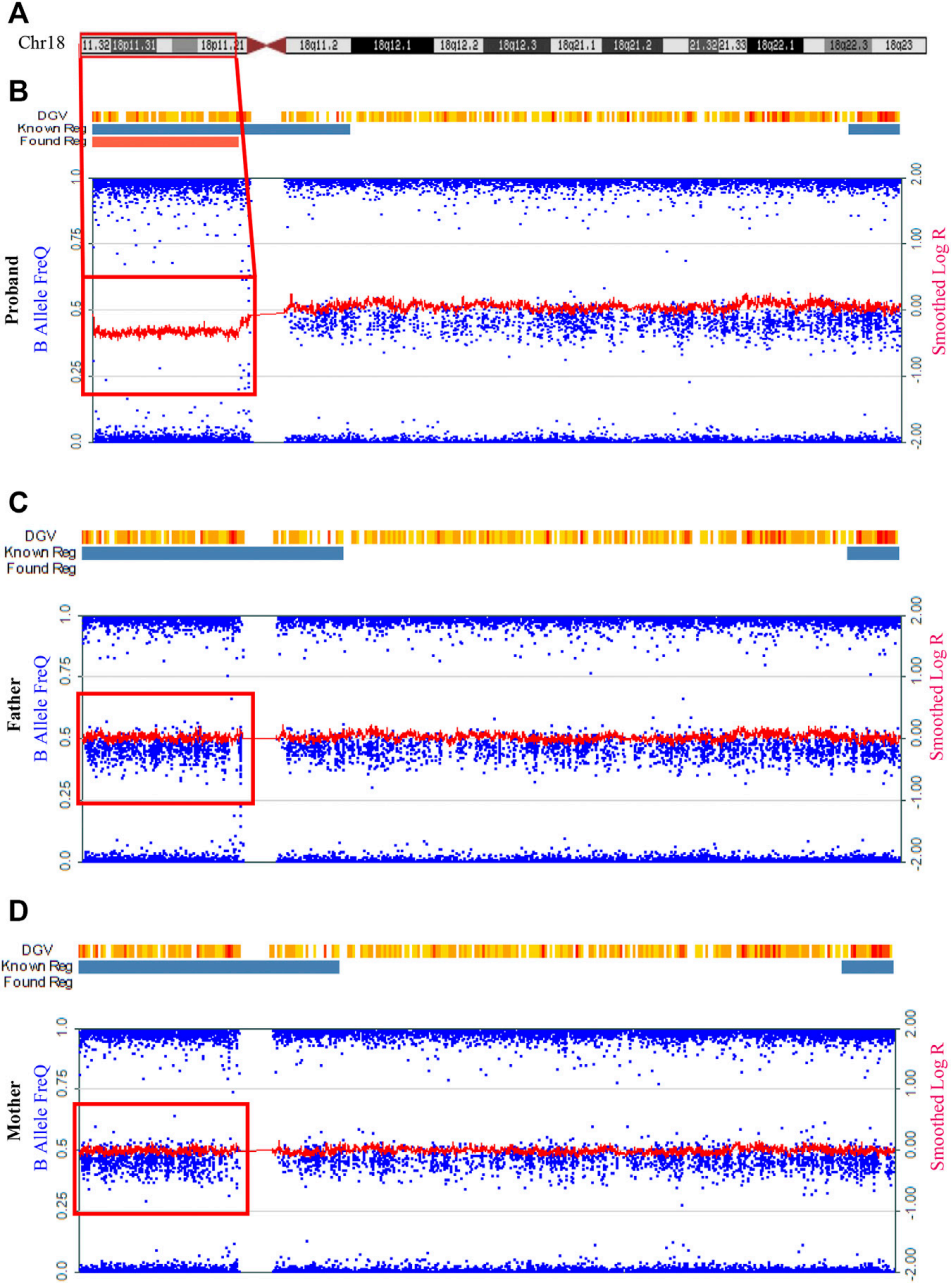
Detection of a 14.3 Mbp heterozygous deletion at chromosome 18p11.32-p11.21 by SNP array in the SMMCI patient. **(A)** Schematic representation of the chromosome 18. **(B–D)** Analysis of the peripheral blood on the Infinium Global Screening Array for the proband **(B)**, his father **(C)** and his mother **(D)**. The red frame indicated the region of chromosome deletion. Blue dots represented “B allele frequency.” Red lines represented “log R ratio.” DGV, Database of Genomic Variants. Known reg and blue bar represented known regions in DGV. Found reg was the abnormal CNV found by the software-red bar represented deletion, green bar represented duplication, and gray bar represented the loss of heterozygosity.

SNP array analysis was then performed to investigate the underlying pathogenic mechanism of the SMMCI patient. An approximately 14.3 Mbp heterozygous deletion was identified in chromosome 18 (chr18:85,037-14,378,579, corresponding to 18p11.32p11.21) in the proband (Figures 2A,B), which was classified as pathogenic. No abnormality was found in other chromosomes (Supplementary Figures). While this heterozygous deletion was not detected in the unaffected parents (Figures 2C,D), which was consistent with the phenotype of the family members, indicating that the mutation carried by the proband is a *de novo* mutation.

**FIGURE 3:**
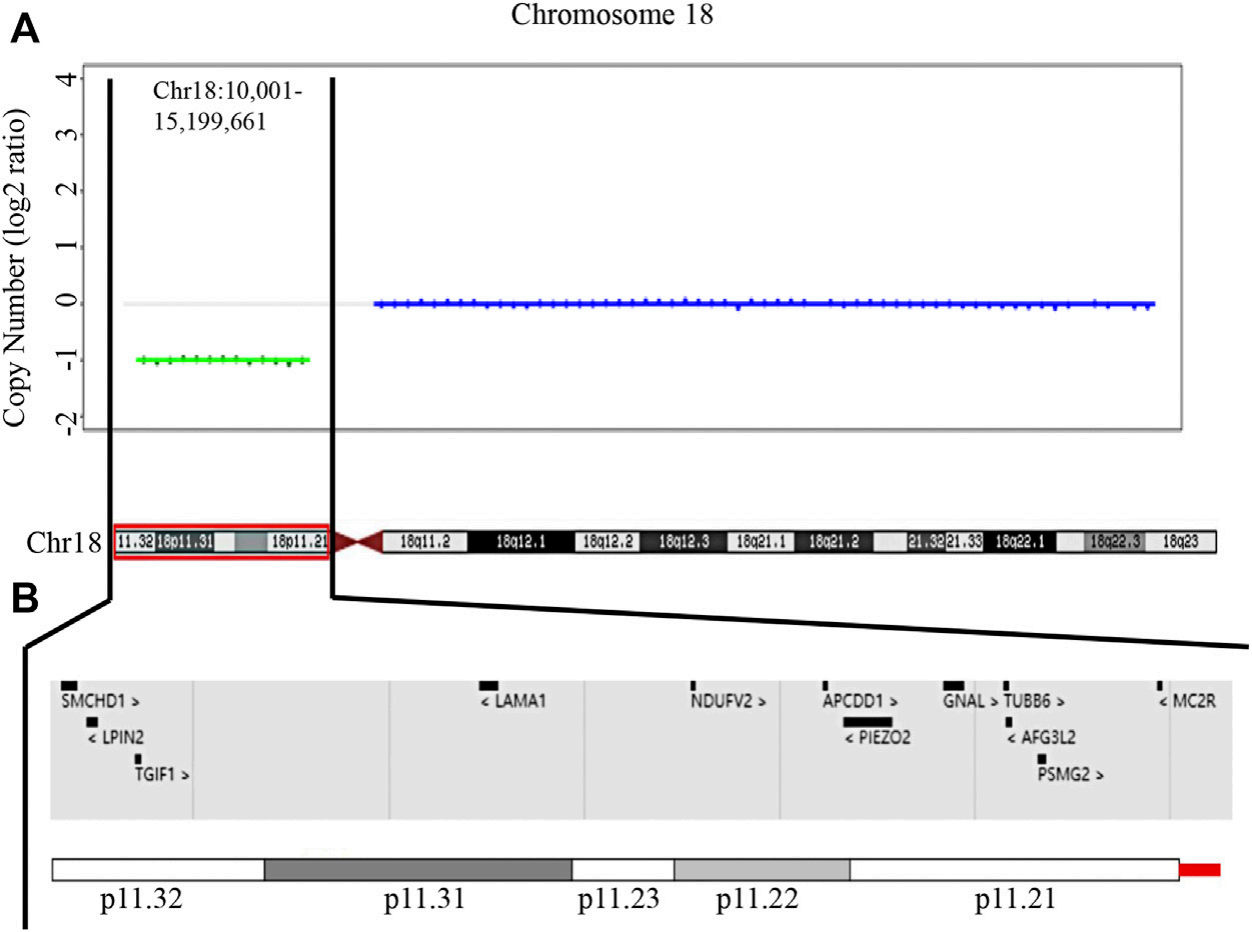
Characterization of the identified 18p deletion analyzed by WGS. **(A)** WGS data showing the proband carries a heterozygous deletion at chromosome 18p11.32-11.21 (chr18:10,001-15,199,661) x1 (h19). **(B)** A view of deleted disease-associated genes in the patient in the DECIPHER database.

To further analyse the molecular characterization of the chromosomal deletion, whole-genome sequencing was further performed. 674,240,728 clean reads were generated, with a genome mapping rate of approximately 99.744%, and the average depth of sequencing was 29.032X. Copy Number (log2 ratio) of chromosome 18p is equal to −1, which represents the heterozygous deletion (Copy Number = 1). Except for chromosome X and Y, the Copy Number (log2 ratio) of the other genomic regions is equal to 0, which represents the normal condition (Copy Number = 2) (Supplementary Figures). Therefore, WGS data showed the proband carried a heterozygous deletion from chr18:10001-15199661, corresponding to chromosome 18p11.32-11.21 (Figure 3A). No abnormality was found in other chromosomes (Supplementary Figures). The whole short arm of chromosome 18 is almost deleted (Figures 3A,B). 194 genes were involved in the region. By retrieving the DECIPHER ((DECIPHER, RRID:SCR_006552)) database, the UniProtKB (UniProtKB, RRID:SCR_004426) database, ClinVar (ClinVar, RRID:SCR_006169) database, and the Online Mendelian Inheritance in Man (OMIM, RRID:SCR_006437) database, we found that 68 were protein-coding genes, 25 were likely dosage-sensitive genes, and 12 had been shown to be associated with diseases. They were structural maintenance of chromosomes flexible hinge domain containing 1 gene (*SMCHD1*), lipin 2 gene (*LPIN2*), TGFB induced factor homeobox 1 gene (*TGIF1*), laminin subunit alpha 1 gene (*LAMA1*), NADH:ubiquinone oxidoreductase core subunit V2 gene (*NDUFV2*), APC down-regulated 1 gene (*APCDD1*), piezo type mechanosensitive ion channel component 2 gene (*PIEZO2*), G protein subunit alpha L gene (*GNAL*), tubulin beta 6 class V gene (*TUBB6*), AFG3 like matrix AAA peptidase subunit 2 gene (*AFG3L2*), proteasome assembly chaperone 2 gene (*PSMG2*), and melanocortin 2 receptor gene (*MC2R*), respectively. The main functions and associated diseases of the above 12 genes were summarized in Table 1. Among them, *TGIF1* mutation has been reported to cause SMMCI or HPE (Gripp et al., 2000). TGIF1 regulates the NODAL/TGF-β signal pathway to maintain the delicate balance between SHH and GLI3 levels (Taniguchi et al., 2012). While there was no report on other 11 genes to cause abnormalities that were similar to the clinical phenotype of SMMCI.

**TABLE 1:** The main functions and associated diseases of the 12 genes in the deletion region of 18p identified by WGS.

Genes	Functions	Diseases
*SMCHD1*	Non-canonical member of the structural maintenance of chromosomes (SMC) protein family, mediating epigenetic silencing by regulating chromatin architecture	Facioscapulohumeral muscular dystrophy 2 (FSHD2) and Bosma arhinia microphthalmia syndrome (BAMS)
*LPIN2*	Magnesium-dependent phosphatidate phosphatase enzyme, regulating fatty acids metabolism and lipid metabolism	Majeed syndrome (MJDS)
*TGIF1*	Active transcriptional corepressor of SMAD2, linking the nodal signaling pathway to the bifurcation of the forebrain and the establishment of ventral midline structures	Holoprosencephaly 4 (HPE4)
*LAMA1*	Mediating the attachment, migration, and organization of cells into tissues during embryonic development by interacting with other extracellular matrix components	Poretti-Boltshauser syndrome (PTBHS)
*NDUFV2*	Core subunit of the mitochondrial membrane respiratory chain NADH dehydrogenase (Complex I)	Mitochondrial complex I deficiency, nuclear type 7 (MC1DN7)
*APCDD1*	Negative regulator of the Wnt signaling pathway, inhibiting Wnt signaling in a cell-autonomous manner	Hypotrichosis 1 (HYPT1)
*PIEZ O 2*	Component of a mechanosensitive channel required for rapidly adapting mechanically activated (MA) currents	Arthrogryposis, distal (DA), Marden-Walker syndrome (MWKS), and Arthrogryposis, distal, with impaired proprioception and touch (DAIPT)
*GNAL*	Modulators or transducers in various transmembrane signaling systems	Dystonia 25 (DYT25)
*TUBB6*	Major constituent of microtubules	Facial palsy, congenital, with ptosis and velopharyngeal dysfunction (FPVEPD)
*AFG3L2*	ATP-dependent protease, mediating axonal and neuron development	Spinocerebellar ataxia 28 (SCA28), Spastic ataxia 5, autosomal recessive (SPAX5), and Optic atrophy 12 (OPA12)
*PSMG2*	Chaperone protein promoting assembly of the 20S proteasome	Proteasome-associated autoinflammatory syndrome 4
*MC2R*	Receptor for corticotropin (ACTH)	Glucocorticoid deficiency 1 (GCCD1)

An extensive literature review had been made and the previously reported chromosomal abnormalities and gene mutations associated with SMMCI were summarized in Tables 2, 3, respectively. The literature search was carried out using the PubMed database (PubMed, RRID:SCR_004846) without temporal limitations. The literature retrieval formula was “[(((solitary median maxillary central incisor) OR (SMMCI)) OR (single central incisor)) OR (single upper central incisor)) OR (a single maxillary incisor].” The retrieved literature were then imported into Endnote X9 software (EndNote, RRID:SCR_014001) for rechecking and duplicate references were removed. By reading the title and preliminarily screening the abstract, we excluded the literature that does not meet the theme. All literature meeting inclusion criteria were carefully read, including the whole text and references. So far, 30 chromosomal structural abnormalities have been reported until now. These include 18p deletion, 7q deletion, 22q deletion, with 18p and 7q deletions being the most frequent ones, 32% (8 out of the 25 individuals) and 28% (7 out of the 25 individuals) respectively. We included the present case in Table 2. Furthermore, 18 different genetic variants were identified in 11 different genes (see Table 3), the majority of which were found in the *SHH* gene. Mutations in this gene have most frequently been associated with SMMCI as they were reported in 5 out of the 12 individuals. In addition, *SIX3* was reported in 3 out of the 12 individuals, *COL4A2* was reported in 2 out of the 12 individuals and *TGIF1* was reported in 1 out of the 12 individuals.

**TABLE 2:** Chromosomal abnormalities associated with SMMCI (not HPE) and its main clinical findings.

Chromosomal abnormality	Main clinical manifestations	Reference
18p deletion	SMMCI, microcephaly, short stature, growth retardation, delayed speech, mild conductive hearing loss	Dolan et al. (1981)
18p deletion	SMMCI, short stature, intellectual disability	Aughton et al. (1991)
18p deletion	SMMCI, anterior nasal stenosis, hypotelorism, growth hormone deficiency, thyroid hormones deficiencies, delayed speech	Hui et al. (1995)
18p deletion	SMMCI, growth hormone deficiency, pituitary dysplasia	Taine et al. (1997)
18p deletion; 15p deletion	SMMCI, microcephaly, short stature, frontal lobes dysplasia, small sella turcica, intellectual disability, delayed speech, alopecia universalis, scoliosis	Kantaputra et al. (2006)
18p11.2 deletion	SMMCI, anterior nasal stenosis, short stature, growth hormone deficiency, ectopic posterior pituitary, delayed speech, absence seizures	Naudi and Fung, (2007)
18p11 deletion	SMMCI, amblyopia, mild intellectual disability	Poelmans et al. (2015)
18p deletion; 4q duplication	SMMCI, short stature, mild intellectual disability, Beckwith–Wiedemann syndrome	
18p11.21 deletion	SMMCI, ptosis, protruding ears	The present study
ring 18	SMMCI, submucous cleft palate, congenital pyriform aperture stenosis, hypotelorism, microcephaly, short stature, growth hormone deficiency	Tavin et al. (1994)
mosaicism ring 18	SMMCI, deviation of nasal septum/narrow nasal cavity, *columella* dysplasia, hypotelorism, microcephaly, short stature, growth hormone deficiency, frontotemporal atrophy, large cisterna magna, intellectual disability, autistic features, fusion of C2–C3 vertebrae, cryptorchidism, small penis	Balci et al. (2011)
		
7q36 deletion	SMMCI, hypotelorism, microcephaly, short stature, growth retardation, intellectual disability	Masuno et al. (1990)
7q36 deletion	SMMCI, hypotelorism, esotropia, microcephaly, short stature, growth retardation, severe intellectual disability, scoliosis	
7q36 deletion	SMMCI, microcephaly, growth retardation	Frints et al. (1998)
7q36 deletion	SMMCI, choanal stenosis, microcephaly, mild intellectual disability	
7q deletion	SMMCI, microcephaly, hypertrophy of tonsil, nasal polyp	Moog et al. (2001)
7q deletion	SMMCI, lumbosacral dysplasia, subcutaneous lumbosacral mass	Tubbs and Oakes (2004)
7q36 deletion; 5q duplication	SMMCI, choanal atresia, hypotelorism, ptosis, microcephaly, short stature, severe intellectual disability, small penis	Poelmans et al. (2015)
		
22q11 deletion	SMMCI, midnasal stenosis, hypotelorism, microcephaly, short stature, Velocardiofacial syndrome (velopharyngeal incompetence)	Hall et al. (1997)
22q11.2 deletion	SMMCI, deviation of nasal septum/narrow nasal cavity, DiGeorge syndrome	Yang et al. (2005)
22q11 deletion	SMMCI, Velocardiofacial syndrome, obstructive sleep apnea	Oberoi and Vargervik (2005)
22q11 deletion	solitary median mandibular central incisor, cleft palate, Velocardiofacial syndrome	
		
47,XXX	SMMCI, bifid uvula, hypotelorism, intellectual disability, epilepsy, patent ductus arteriosus	Miura et al. (1993)
		
1q duplication; 6q deletion	SMMCI, hypertelorism, microcephaly, growth retardation, corpus callosum dysgenesis, intellectual disability, seizures	Chen et al. (2012)
		
1p31.3 duplication	SMMCI, deviation of the nasal septum, delayed myelin degeneration, deep sulci in cerebral hemispheres, delayed speech, intellectual disability, epilepsy	Yu et al. (2021)
2q21.2 deletion; 20p12.1 duplication	SMMCI, hypertelorism, convergent strabismus, short stature, growth hormone deficiency, growth retardation, empty sella, panhypopituitarism, mild intellectual disability, hypothyroidism, absence of puberty, inner genitals dysplasia	Szakszon et al. (2012)

**TABLE 3:** Sequence variations found in SMMCI (not HPE) and its main clinical findings.

Gene	Nucleotide variation	Main clinical manifestations	Reference
*SHH*	c.331A > T p.I111F	SMMCI, choanal stenosis	Nanni et al. (2001)
*SHH*	c.331A > T p.I111F	SMMCI, choanal stenosis, slow learner	
*SHH*	c.995T > C p.V332A	SMMCI, choanal stenosis, hypotelorism, microcephaly, patent ductus arteriosus, premaxillary region dysplasia	Dubourg et al. (2004)
*SHH*	c.995T > C p.V332A	SMMCI, cleft palate, hypotelorism, short stature, corpus callosum dysplasia, colpocephaly	
*SHH*	c.420C > G p.H140Q	SMMCI, hypotelorism, microcephaly, neurohypophyseal tumor	Ribeiro and Richieri-Costa (2005)
*SIX3*	c.686C > T p.P229L	SMMCI, hypotelorism	El-Jaick et al. (2007)
*SIX3*	c.109G > T p.G37C	SMMCI, cleft lip/palate, choanal atresia, ptosis, coloboma, microcephaly, short stature, mild intellectual disability, ventricular septal defect	Poelmans et al. (2015)
*TGIF1*	c.83C > G p.S28C	SMMCI, congenital nasal pyriform aperture stenosis, hypotelorism, microcephaly, growth retardation, corpus callosum dysplasia	Gripp et al. (2000)
*COL4A2*	c.3896G > A p.G1299E	SMMCI, congenital nasal pyriform aperture stenosis, microcephaly, growth retardation, schizencephaly, dermoid cyst	Gazdagh et al. (2017)
*COL4A2*	c.3896G > A p.G1299E	SMMCI, delayed speech, dermoid cyst	
*DISP1*	c.4049delC p.S1350fs	SMMCI, choana stenosis, coloboma of iris and retina, microcephaly, growth hormone deficiency, growth retardation, corpus callosum dysplasia, delayed speech, epilepsy, central diabetes insipidus	Garcia Rodriguez et al. (2019)
*ZIC2*	c.80C > T p.A27V		
*PTCH1*	c.109G > T p.G37W		
*SIX3*	c.514G > A p.A172T		
*ASLX1*	c.583G > A p.A195T		
*SMO*	c.1265G > A p.G422E	SMMCI, congenital nasal pyriform aperture stenosis	Zatonski et al. (2020)
*PLD2*	c.956delA p.Q319fs		
*P2RY13*	c.615G > A p.W205*		

## Discussion

In the study, a heterozygous deletion at the chromosome 18p region that includes the *TGIF1* gene was identified in one Chinese family with a typical SMMCI phenotype. The identified chromosomal variant was not detected in the unaffected parents, indicating it was a *de novo* mutation.

SMMCI is a complex disorder consisting of multiple, mainly midline defects of development resulting from unknown factor(s) operating in the uterus about the 35th-38th day(s) from conception (Hall et al., 1997). However, the clear pathogenesis of SMMCI remains uncertain. The odontogenic epithelium appeared on the 35th day in the uterus and it diffused medially and distally to form the maxillary dental lamina (Hall et al., 1997). It was estimated that at this time the normal lateral growth of the maxillae and orbits, together with the other midline structures in the region appears to have slowed or ceased for reasons unknown, leading to the left and right dental laminae fuse prematurely in the midline, thereby preventing the normal formation of the two tooth germs for the left and right central incisors and their intervening bone and soft tissue (Nanni et al., 2001). The SMMCI tooth is precisely located in the midline of the maxillary and it appears to be of the two distal halves of the left and right central incisor teeth. Therefore, it was considered that the dental lamina had fused prematurely in the midline, resulting in apposition and fusion of the forming tooth buds (Nanni et al., 2001).

SMMCI syndrome has an autosomal condition inheritance; however, its reported genetic pathogenesis is very heterogeneous. A variety of chromosomal structural abnormalities can lead to SMMCI (see Table 2). Deletions on chromosomes 7, 18, and 22 have been reported, with 18p and 7q deletions being the most frequent ones. Consistent with previous reports, a heterozygous deletion at the chromosome 18p region was identified by SNP array and confirmed by WGS in the present study. It is not hard to explain because this chromosomal region (at 18p11.31) harbors the HPE gene, TGIF1 (Gripp et al., 2000). SMMCI patients combined with chromosomal abnormalities typically presented with microcephaly, short stature, and (or) growth hormone deficiency. In addition, hypotelorism, delayed speech, and intellectual disability were also shown in some patients. Wester et al. (2006) had summarized seven patients’ clinical symptoms combined with 18p deletion and found that the clinical phenotypes of the patients varied greatly due to the size and breakpoints of deletion. In addition, different phenotypes could be shown with the same genotype. One of these reported cases with the same cytogenetic aberrations as the proband in the present study showed a quite different phenotype with the proband, with hypertelorism and muscular hypotonia and ptosis, but absent SMMCI (Wester et al., 2006). The relationship between genotype and phenotype of SMMCI still needs further exploration.

In addition, some other genetic variants were identified in SMMCI (see Table 3). Heterozygous mutations in the *SHH* on chromosome 7q36.3 have most frequently been associated with SMMCI, 41.7% (5 out of the 12 individuals). Furthermore, some genetic variants in other different genes, including *SIX3*, *TGIF1*, *COL4A2*, *DISP1*, *ZIC2*, *PTCH1*, *ASLX1*, *SMO*, *PLD2*, and *P2RY13*, have also been detected in SMMCI. In the present study, a 14.3 Mbp heterozygous deletion at chromosome 18p11.32-p11.21 was identified in the proband and 194 genes were involved in the chromosome region. Among them, 12 genes had been shown to be associated with diseases, including *TGIF1*. *TGIF1* mutation had been detected in SMMCI or holoprosencephaly (HPE) patients (Gripp et al., 2000). TGIF1 regulates the NODAL signal pathway, thereby maintaining an appropriate balance between SHH and GLI3 levels (Taniguchi et al., 2012). It is widely reported that defects on SHH protein or in its signal pathway could lead to HPE. While the relationship of SMMCI to the genes currently implicated in the pathogenesis of HPE (*SHH*, *ZIC2*, *SIX3*, *TGIF1*, and *DKK1*) is still unclear.

Both the parents of the proband and the proband considered that the quality of life was not affected by the phenotype. However, SMMCI is a risk factor of HPE. Therefore, genetic counseling and prenatal diagnosis were recommended for the patient and his family members in the future. As for the SMMCI tooth, orthodontic treatment combined with prosthetic treatment may be a reasonable option for the long-term treatment when the patient is an adult.

In summary, a heterozygous deletion at the 18p chromosomal region that includes the *TGIF1* gene was identified in one Chinese patient with a typical SMMCI phenotype. Our finding indicates that the *de novo* 18p deletion resulted in the SMMCI phenotype in the present study. Our results provide new genetic evidence that structural abnormality in chromosome 18p contributes to SMMCI. Our finding extends the genetic spectrum of SMMCI and might contribute to the genetic diagnosis and genetic counseling of families with SMMCI. However, there are 194 genes in this chromosomal region. Whether other genes are involved in the regulation of craniomaxillofacial development still needs further exploration.

## Data Availability

The datasets presented in this study can be found in NCBI Sequence Read Archive with the accession number PRJNA764834.
