# Supplementary material for: Solitary Median Maxillary Central Incisor Syndrome: An Exploration of the Pathogenic Mechanism
Source: Front Genet. 2022 Jan 24;13:780930. doi: 10.3389/fgene.2022.780930 (PMC8819842; doi:10.3389/fgene.2022.780930)
Supplement: Supplementary file 1 [file DataSheet1.DOCX]

Supplementary Material

# Supplementary Figures


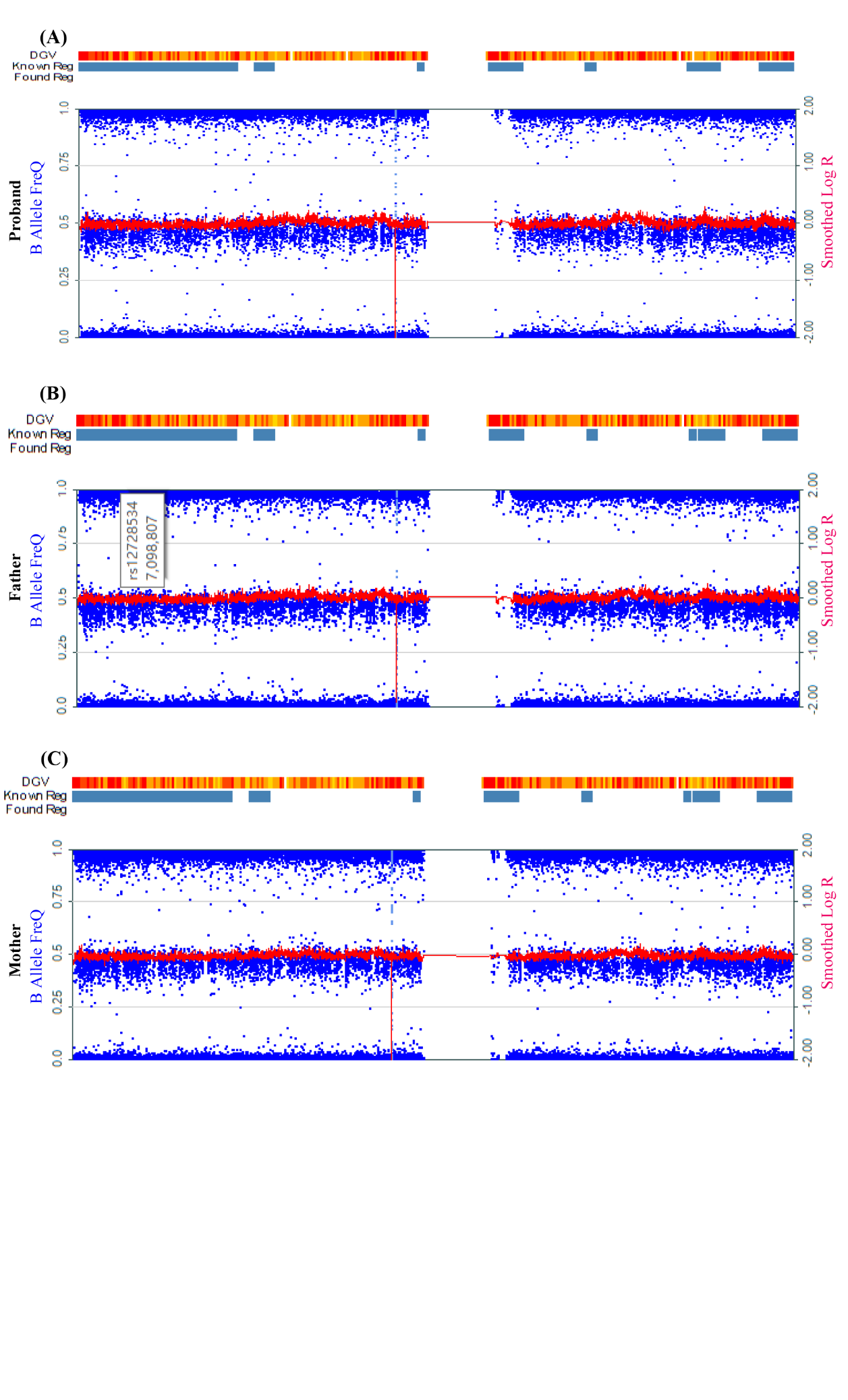


**Supplementary Figure 1.** No abnormalities were detected in chromosome 1.


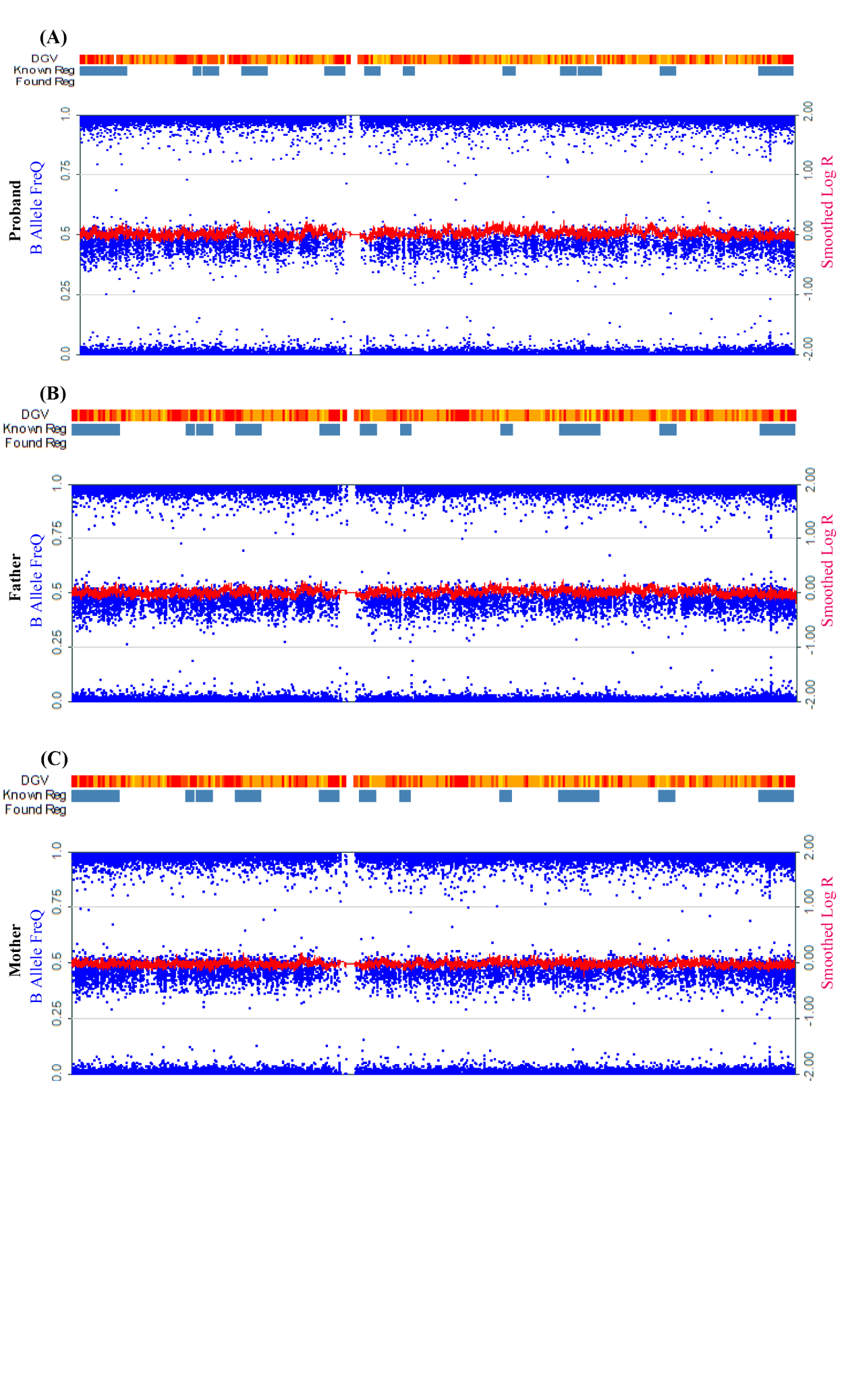


**Supplementary Figure 2.** No abnormalities were detected in chromosome 2.


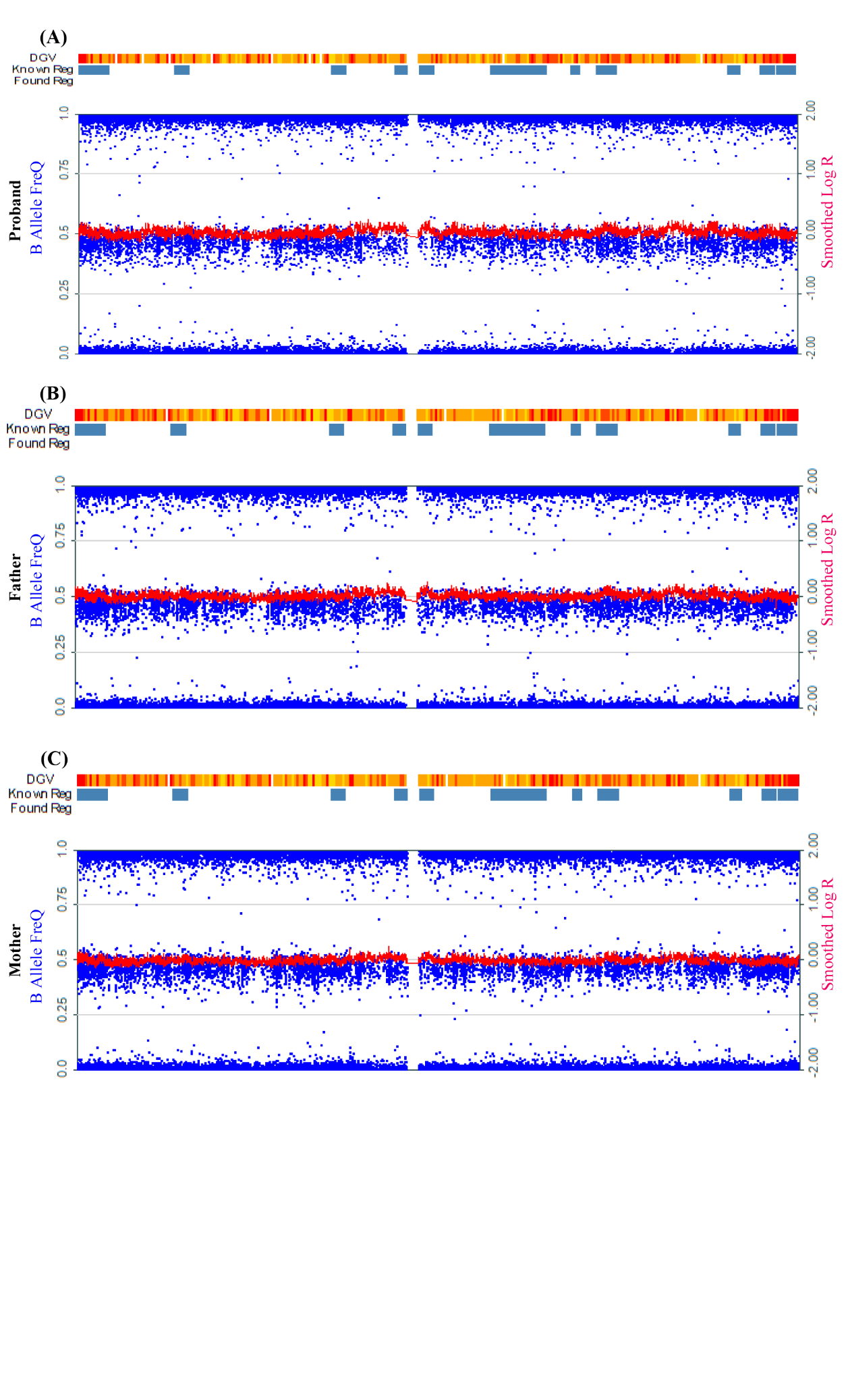


**Supplementary Figure 3.** No abnormalities were detected in chromosome 3.


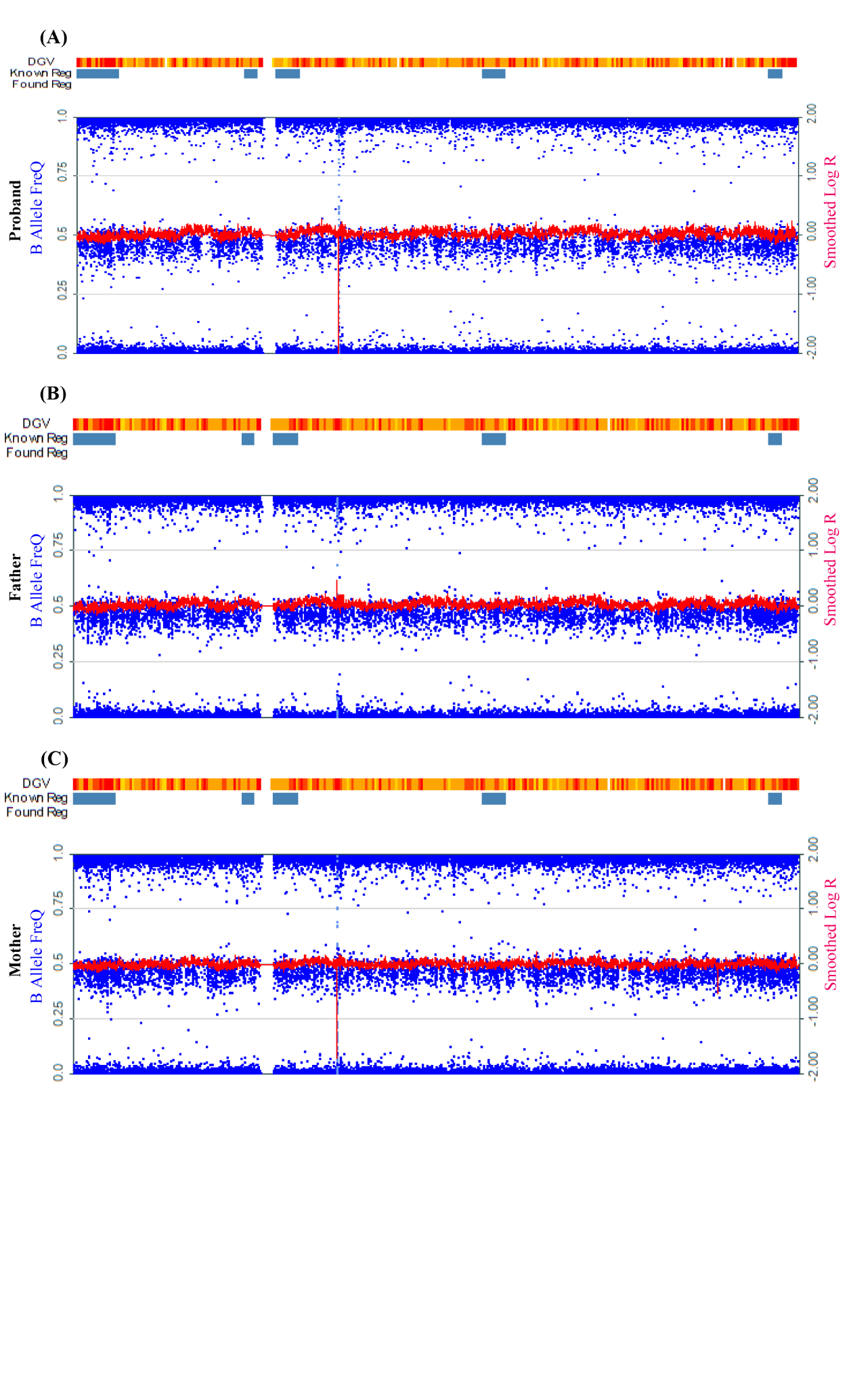


**Supplementary Figure 4.** No abnormalities were detected in chromosome 4.


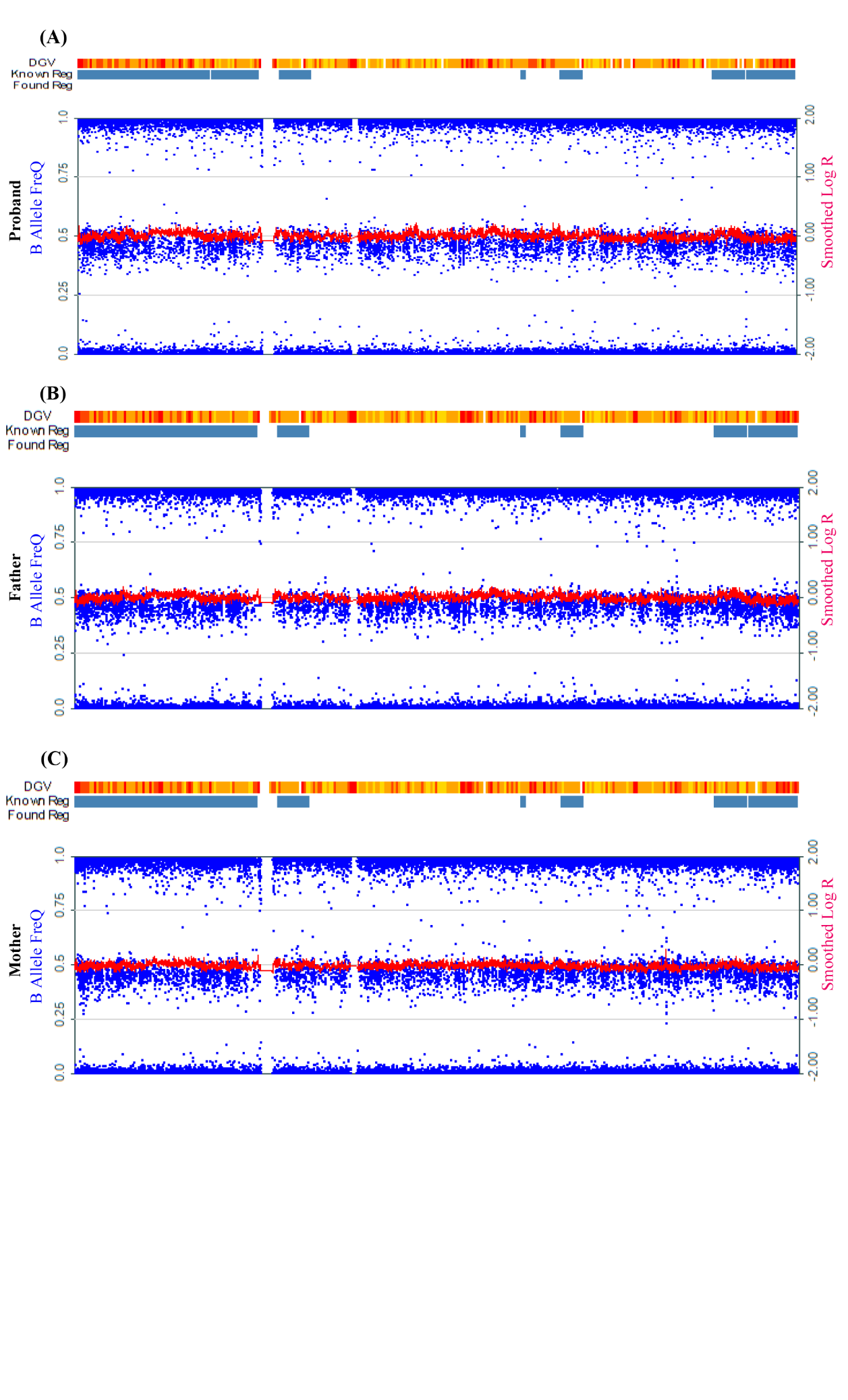


**Supplementary Figure 5.** No abnormalities were detected in chromosome 5.


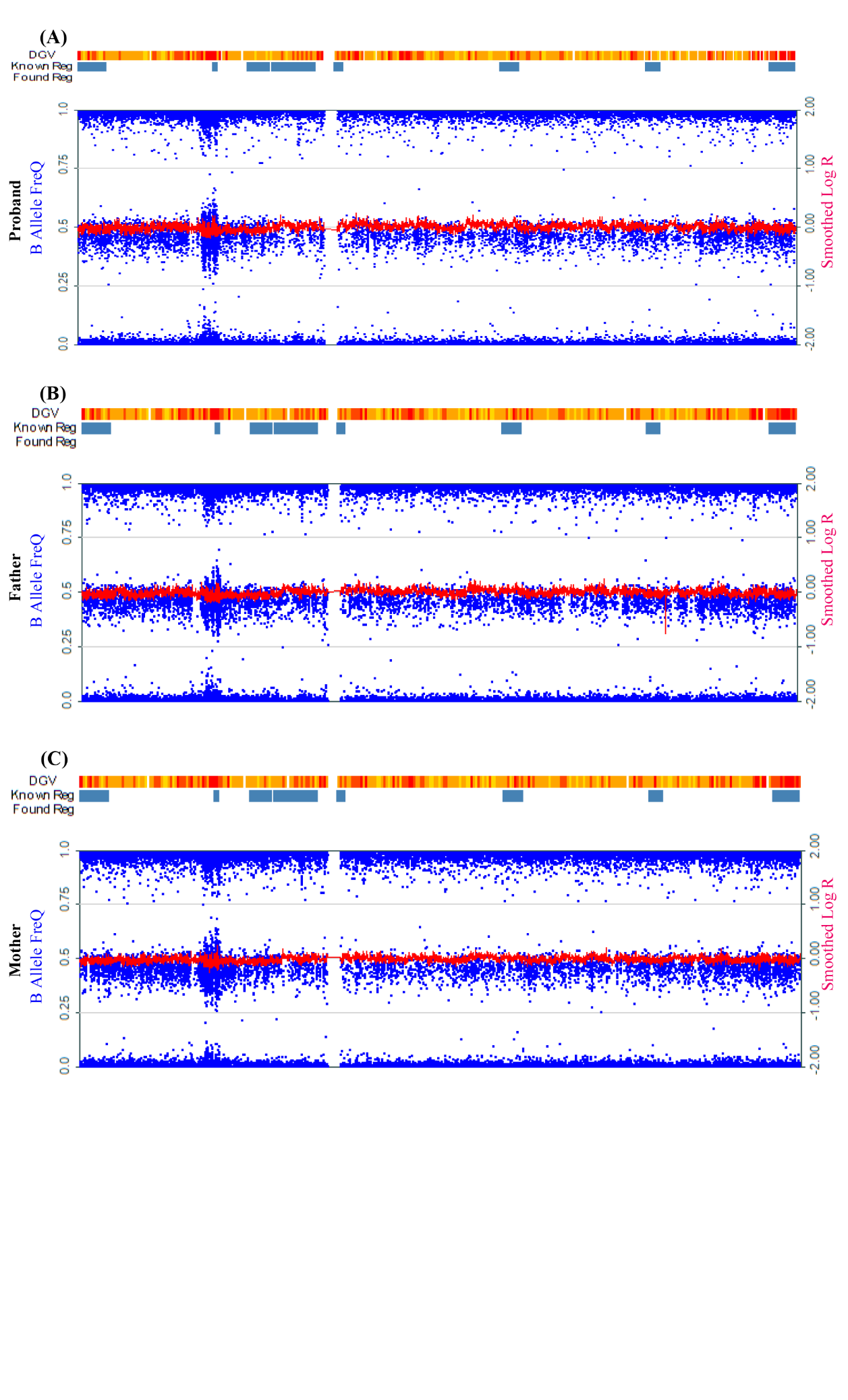


**Supplementary Figure 6.** No abnormalities were detected in chromosome 6.


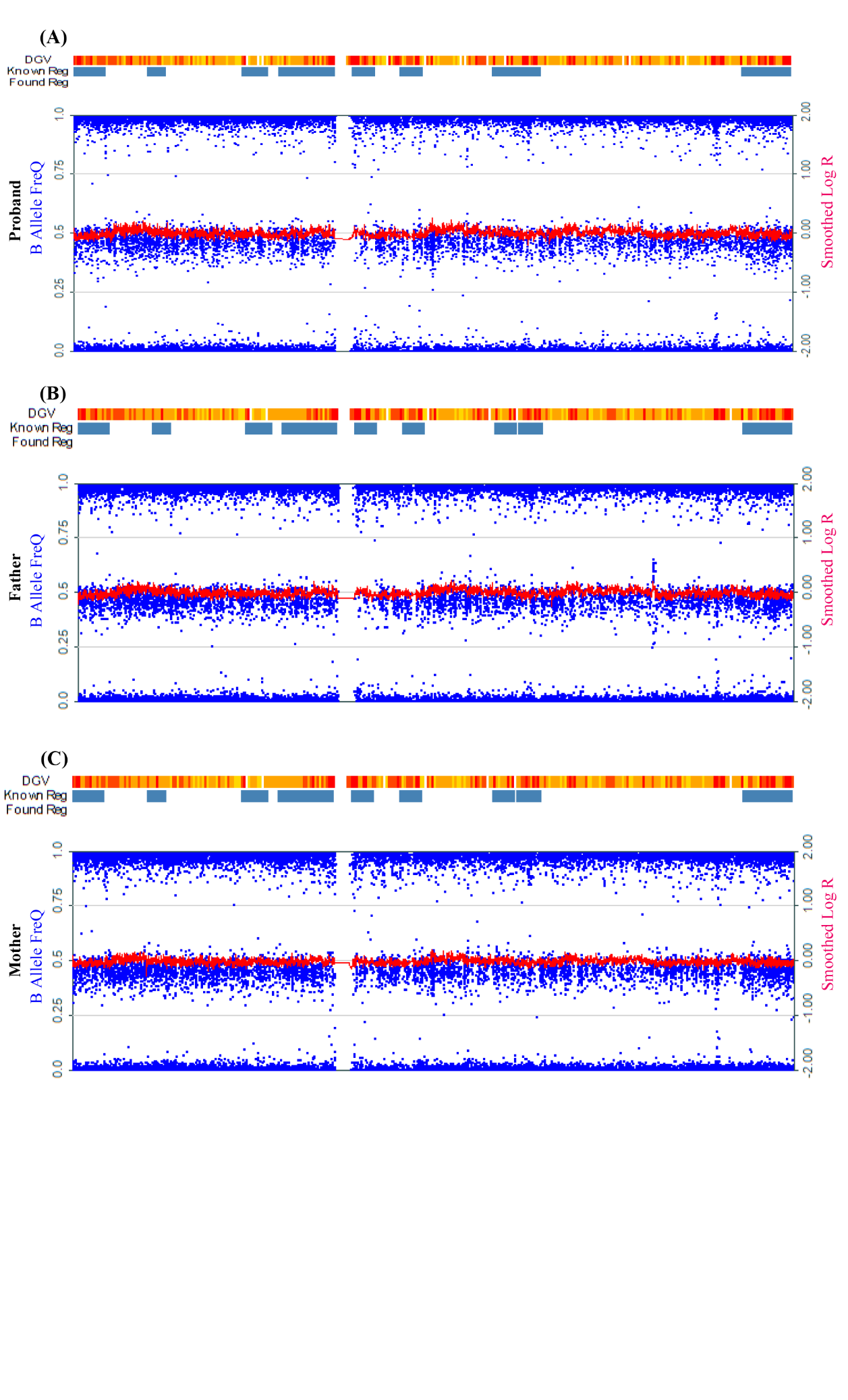


**Supplementary Figure 7.** No abnormalities were detected in chromosome 7.


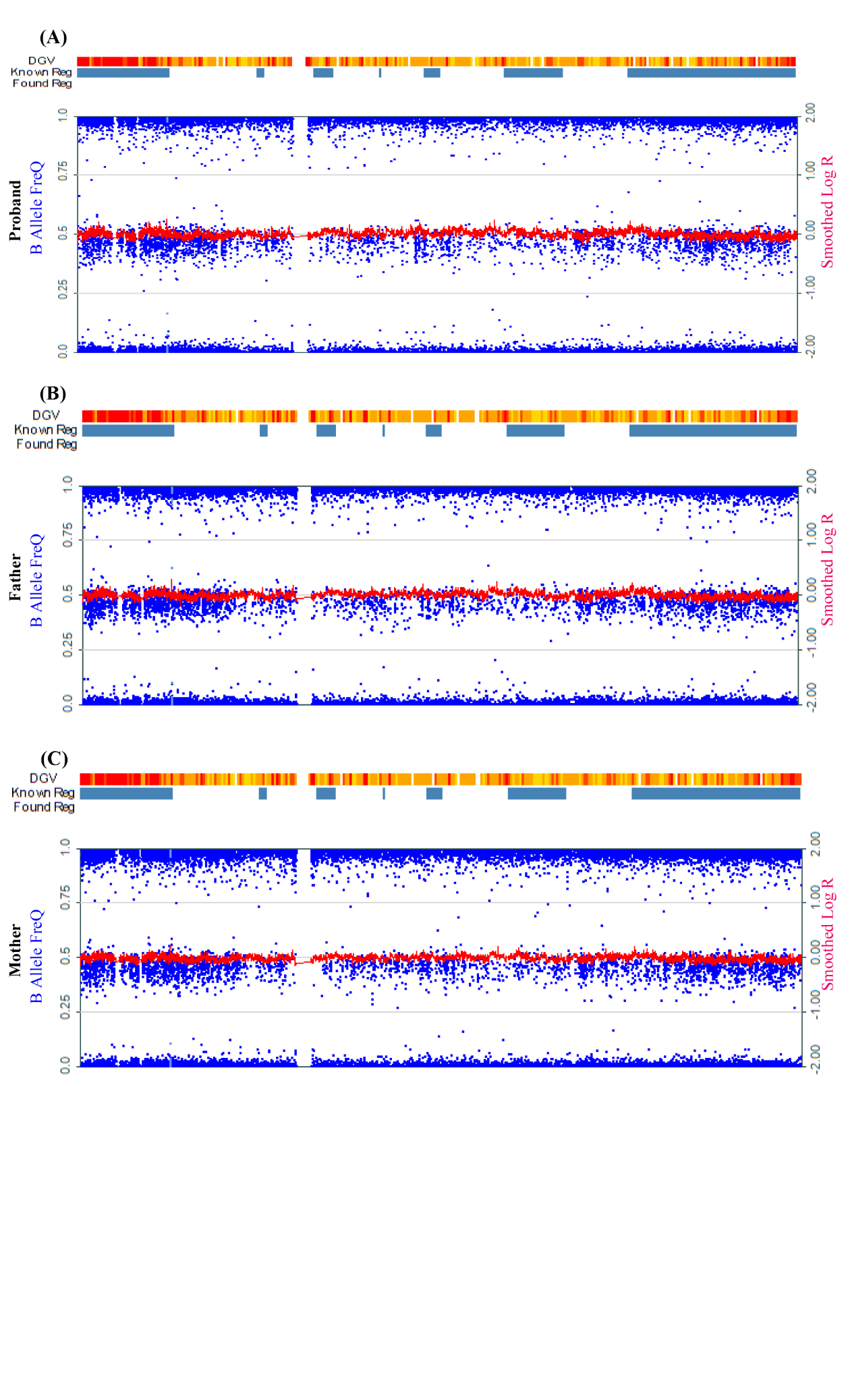


**Supplementary Figure 8.** No abnormalities were detected in chromosome 8.


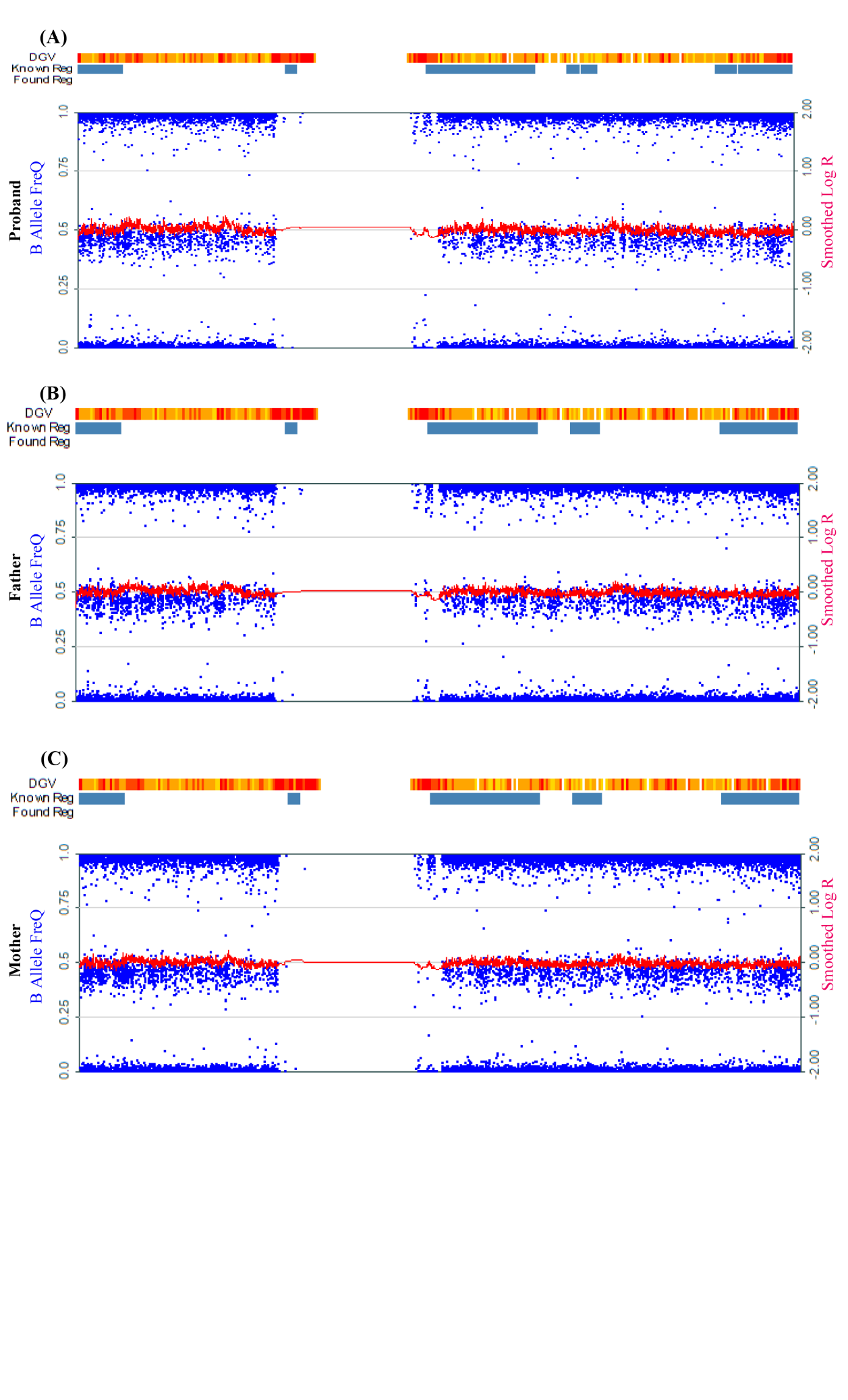


**Supplementary Figure 9.** No abnormalities were detected in chromosome 9.


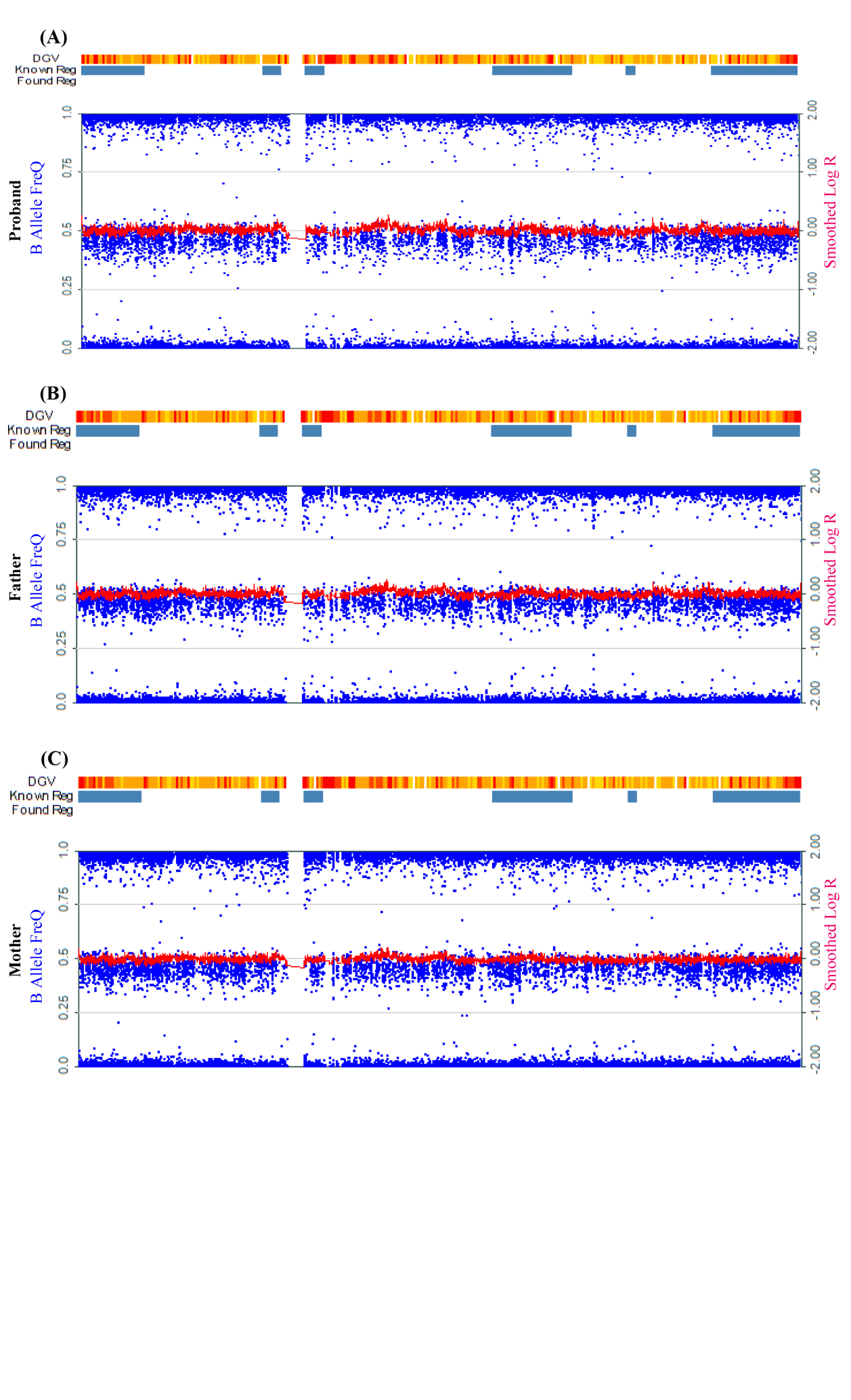


**Supplementary Figure 10.** No abnormalities were detected in chromosome 10.


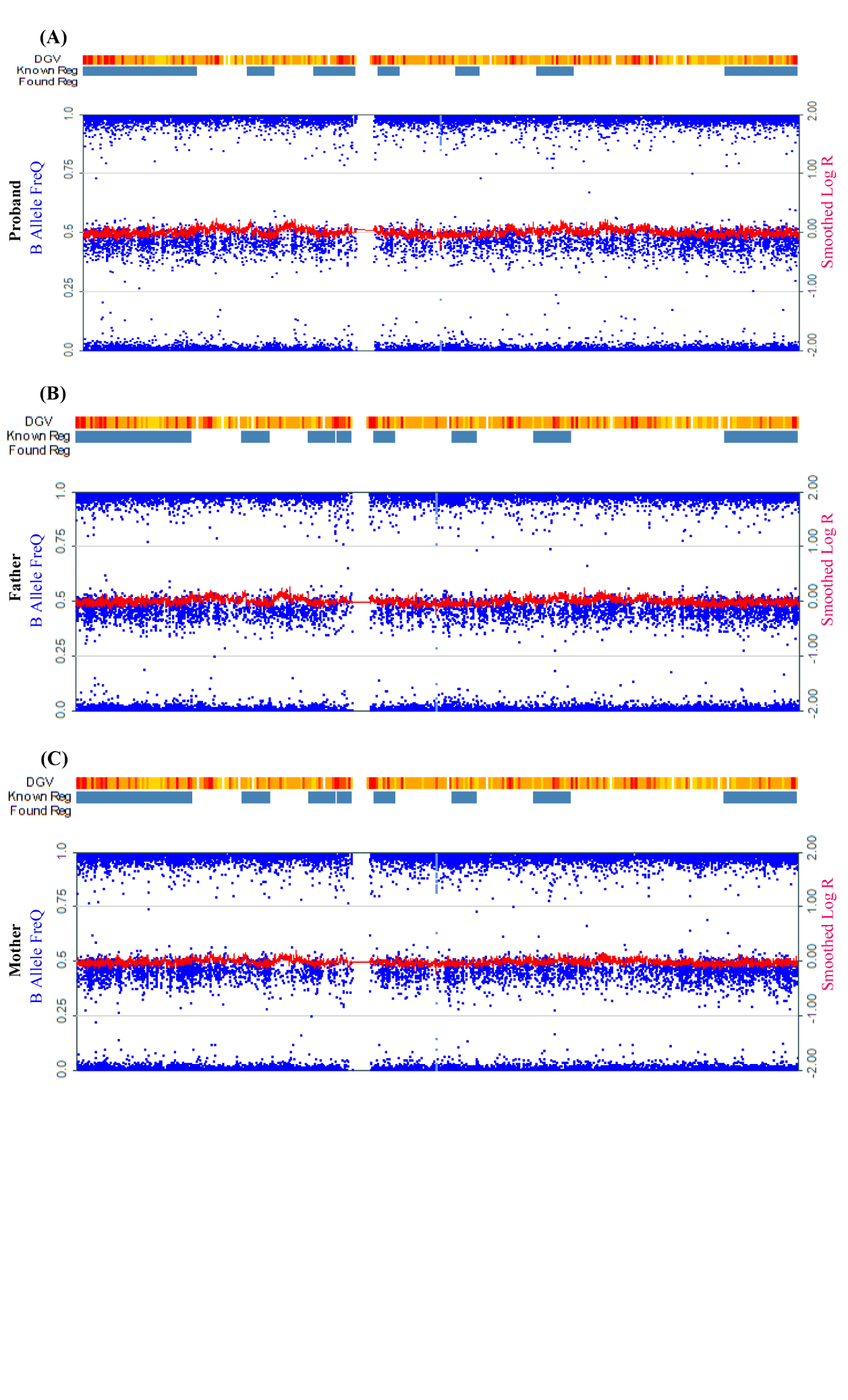


**Supplementary Figure 11.** No abnormalities were detected in chromosome 11.


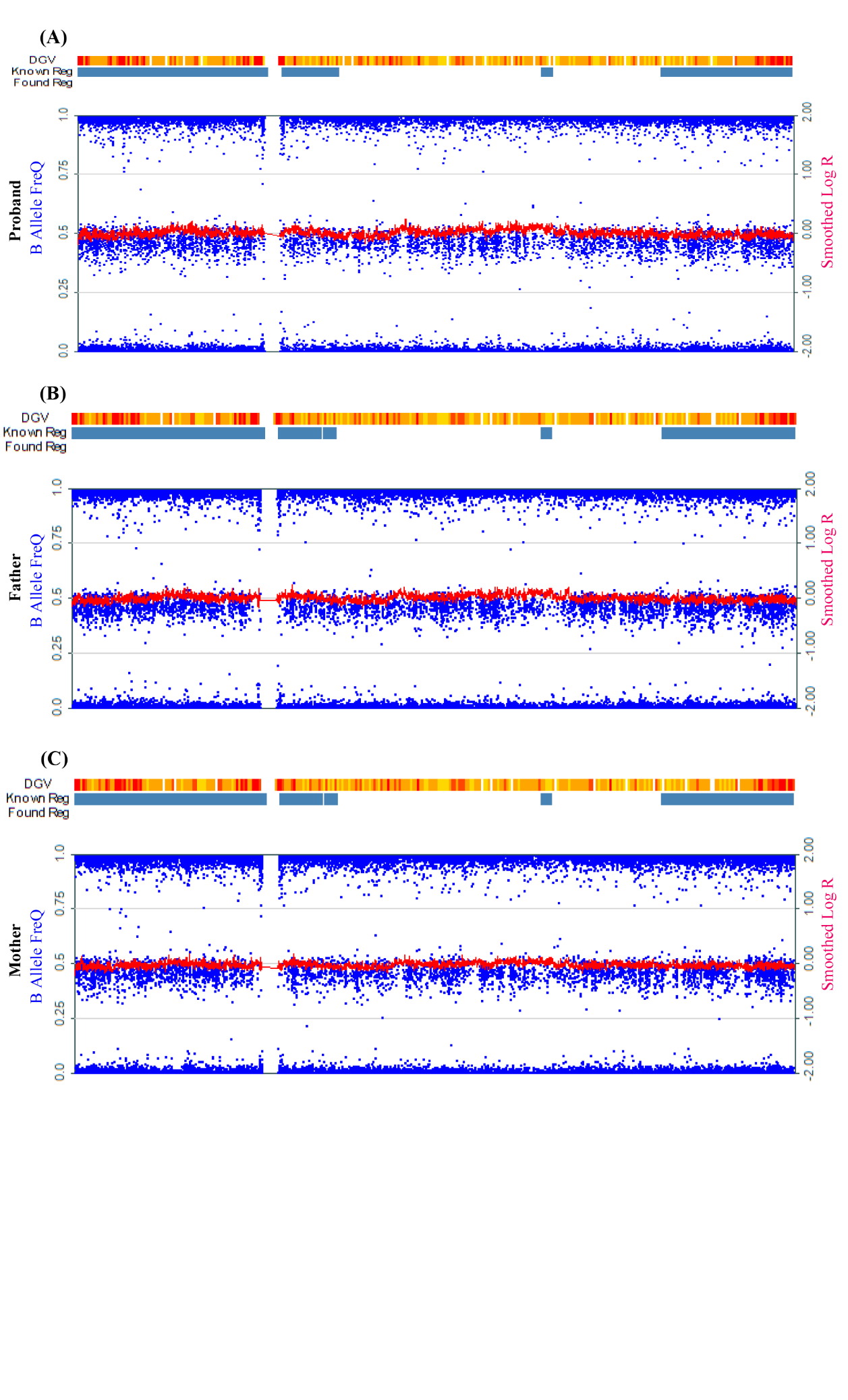


**Supplementary Figure 12.** No abnormalities were detected in chromosome 12.


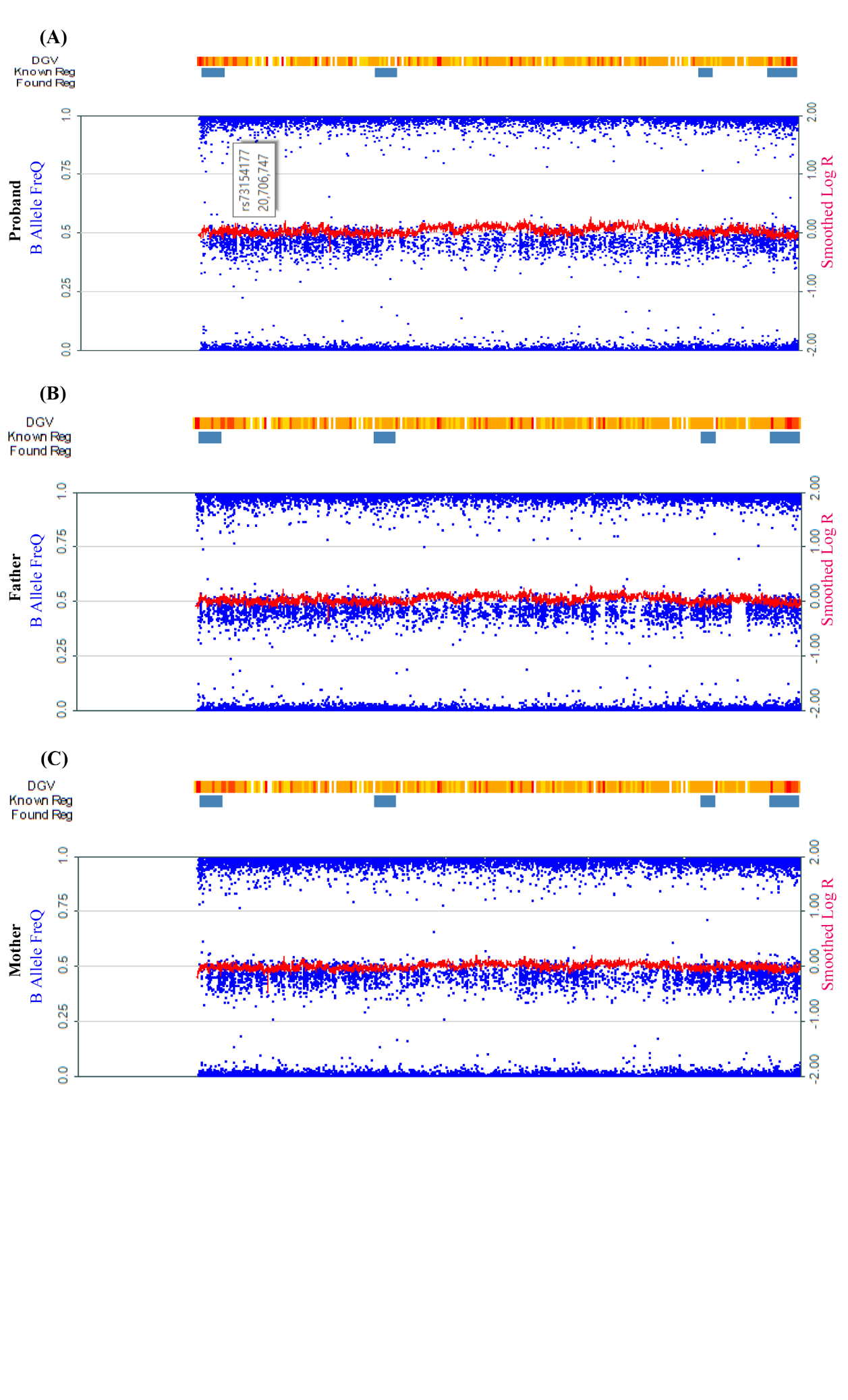


**Supplementary Figure 13.** No abnormalities were detected in chromosome 13.


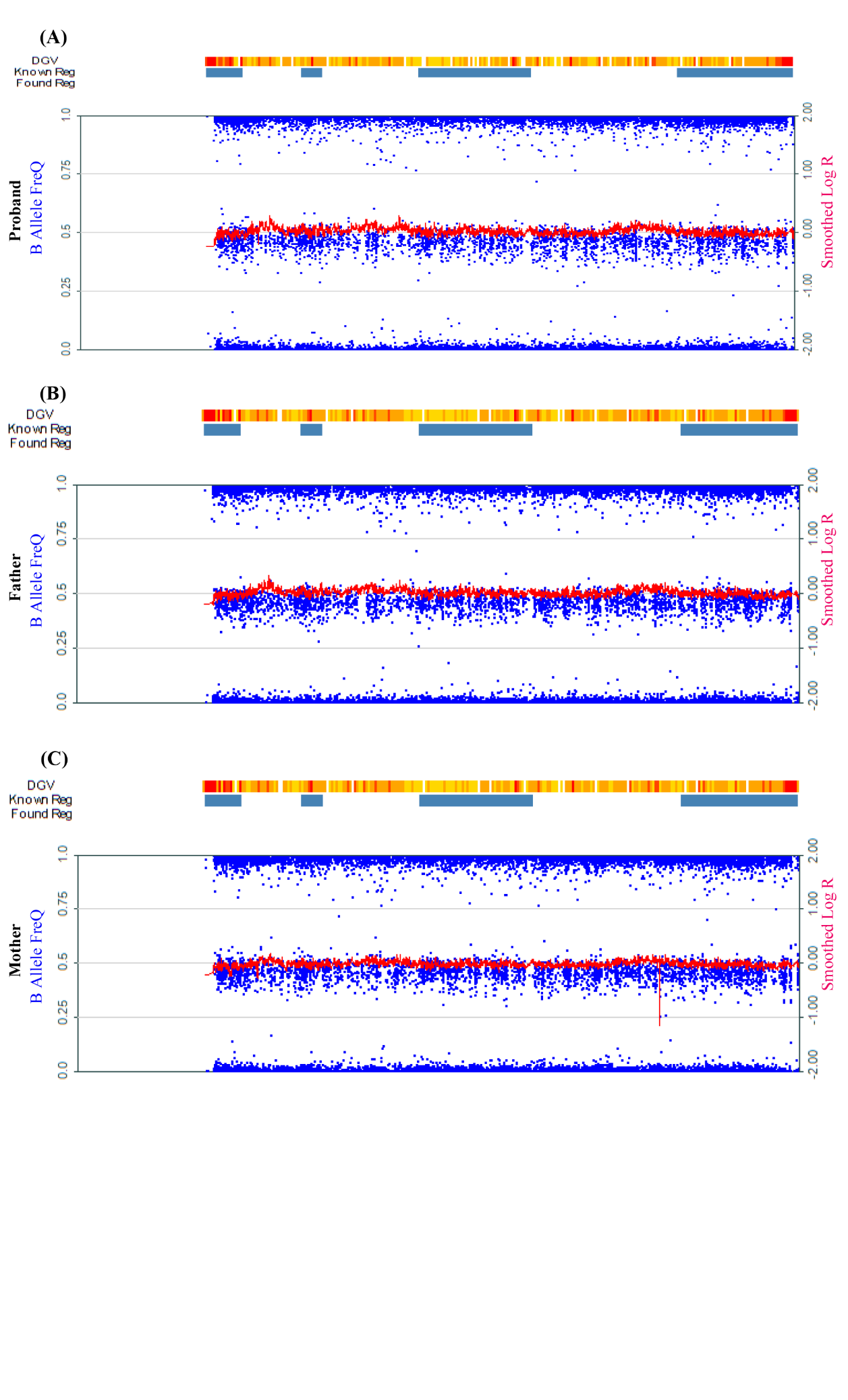


**Supplementary Figure 14.** No abnormalities were detected in chromosome 14.


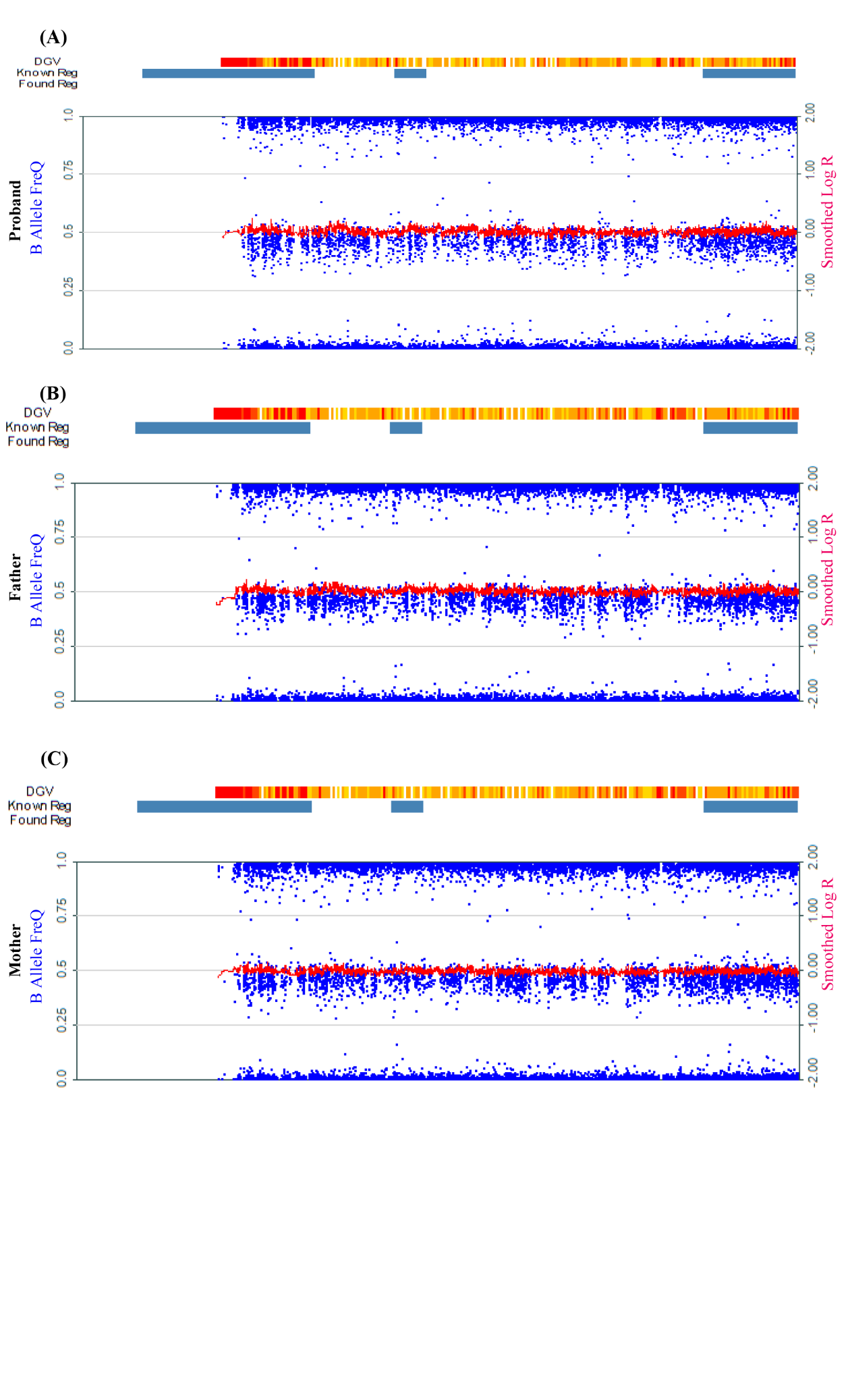


**Supplementary Figure 15.** No abnormalities were detected in chromosome 15.


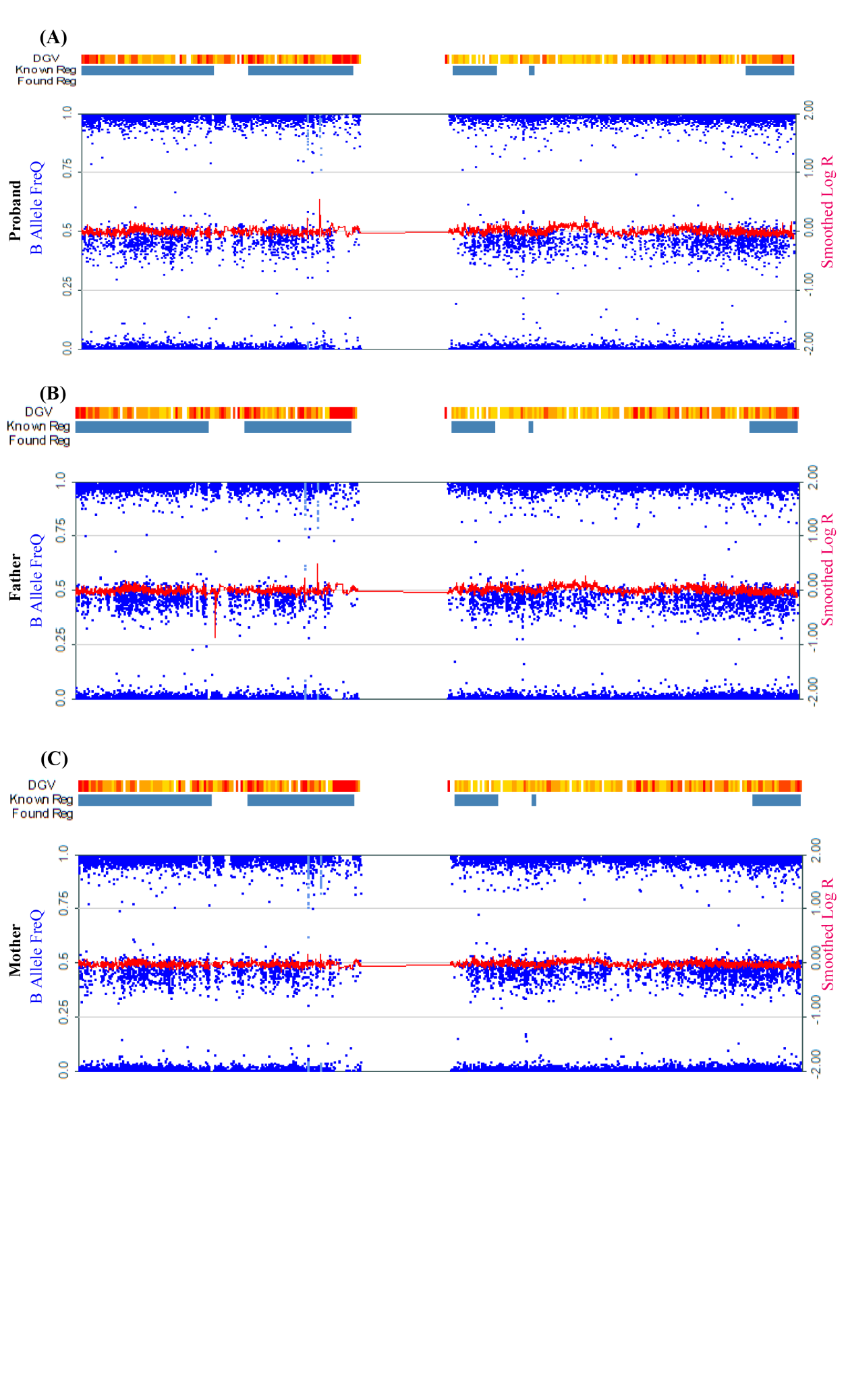


**Supplementary Figure 16.** No abnormalities were detected in chromosome 16.


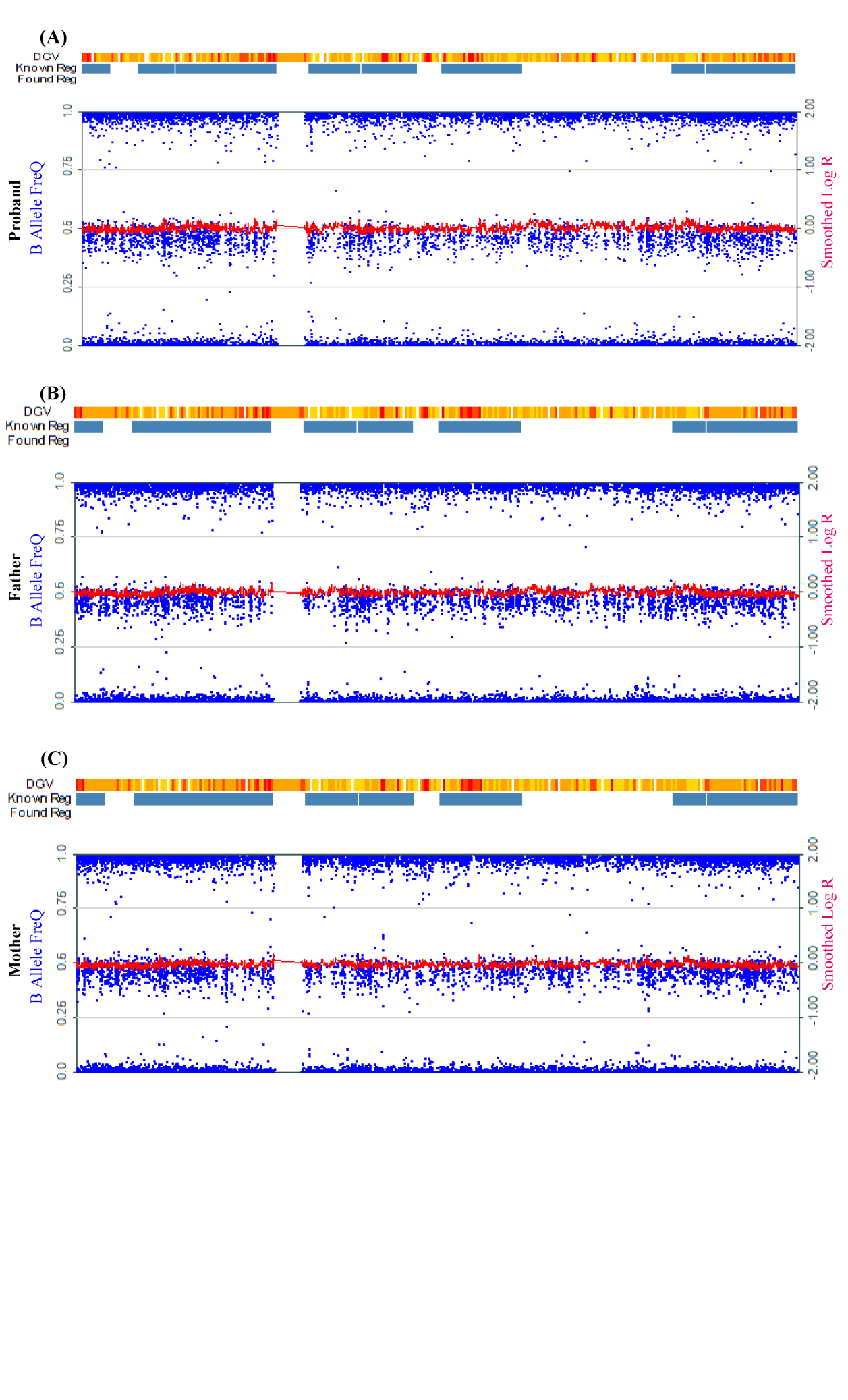


**Supplementary Figure 17.** No abnormalities were detected in chromosome 17.


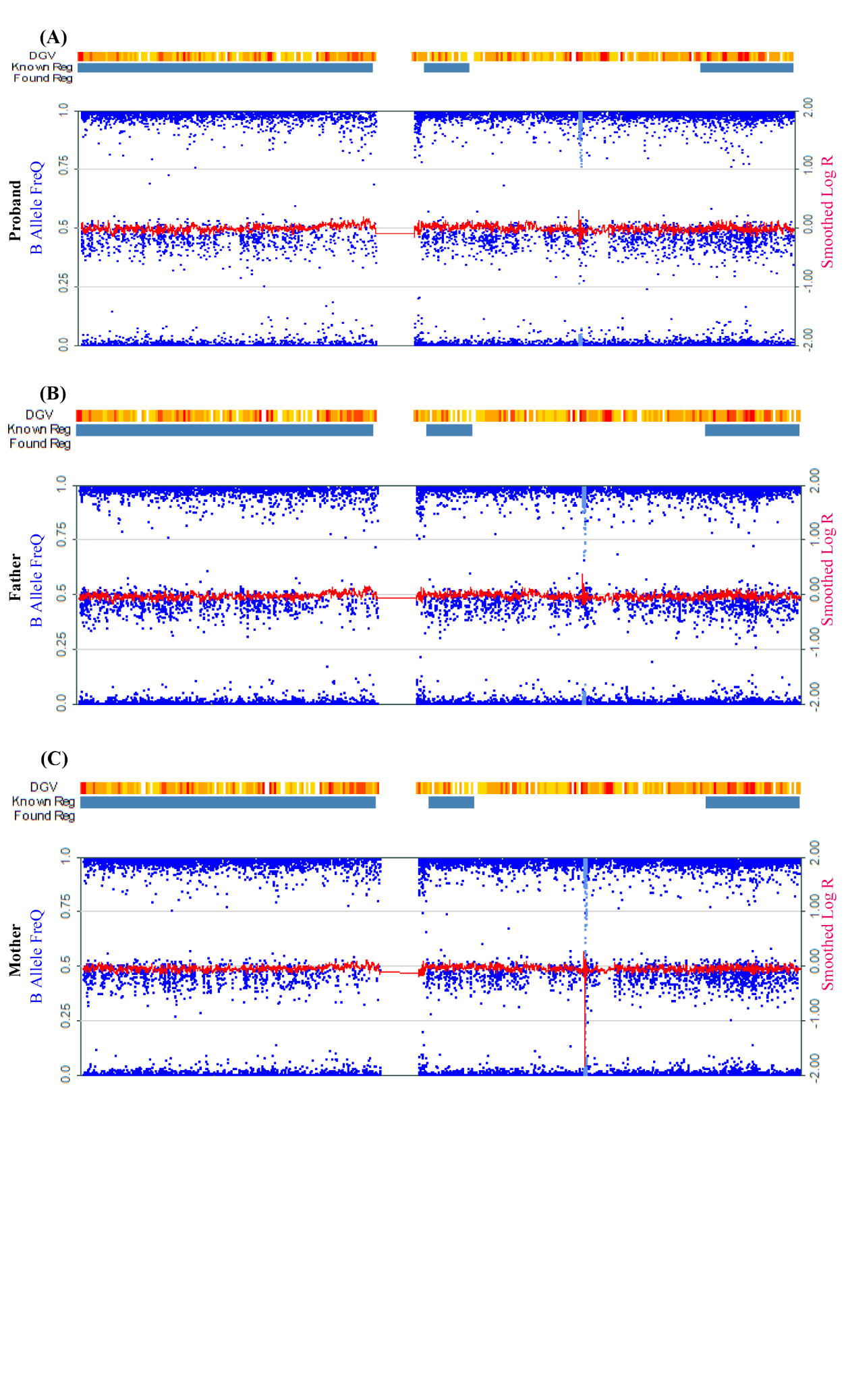


**Supplementary Figure 18.** No abnormalities were detected in chromosome 19.


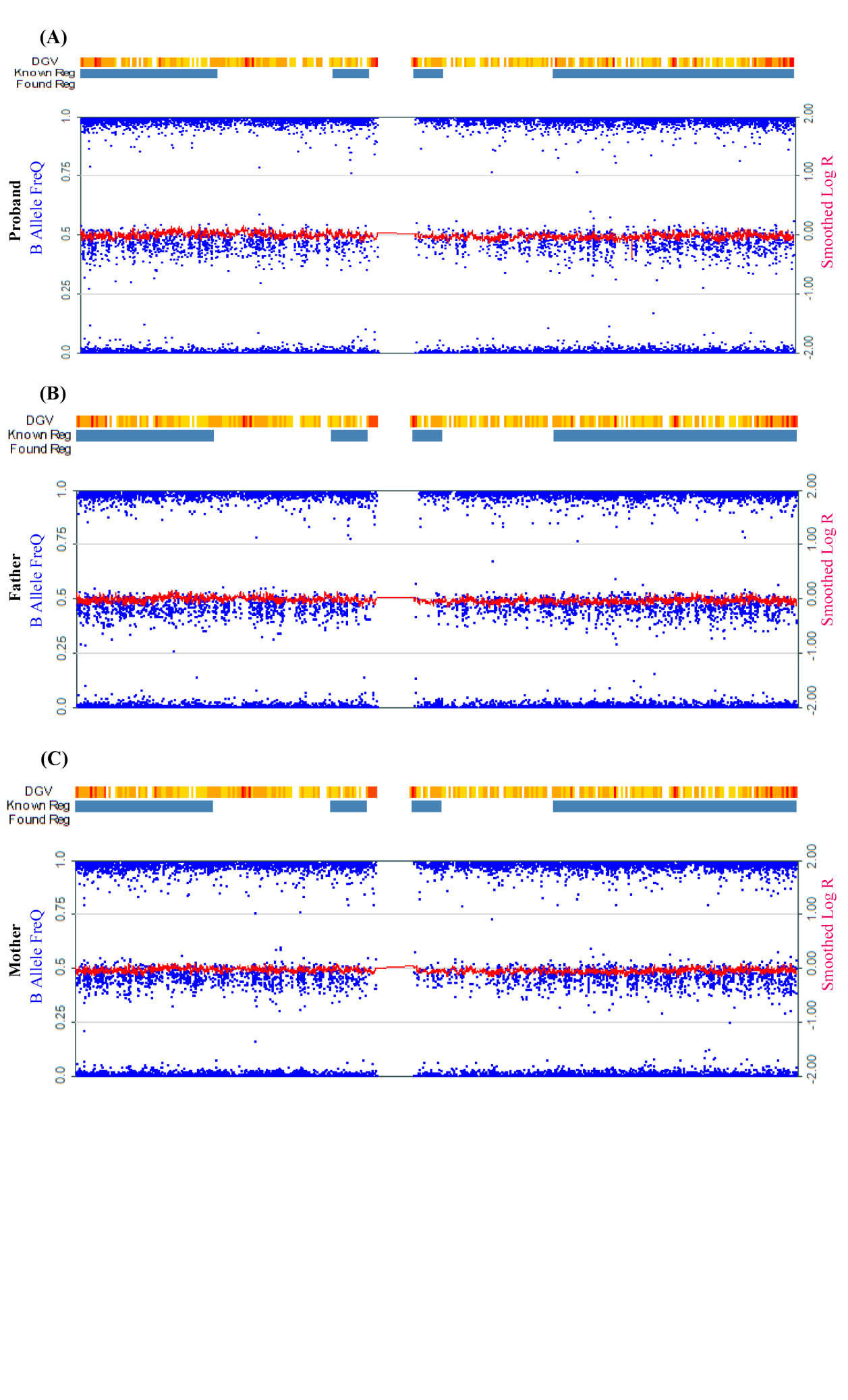


**Supplementary Figure 19.** No abnormalities were detected in chromosome 20.


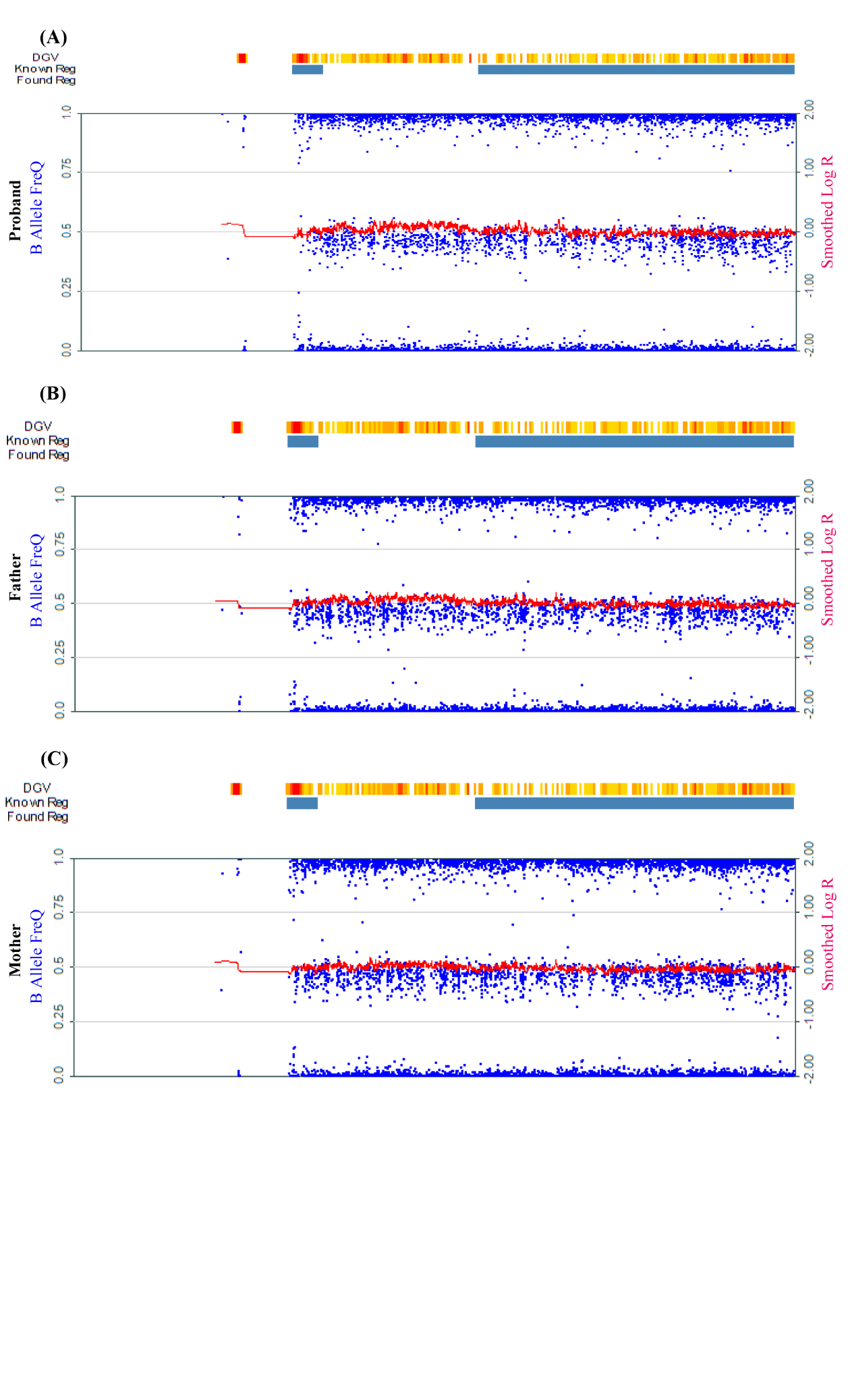


**Supplementary Figure 20.** No abnormalities were detected in chromosome 21.


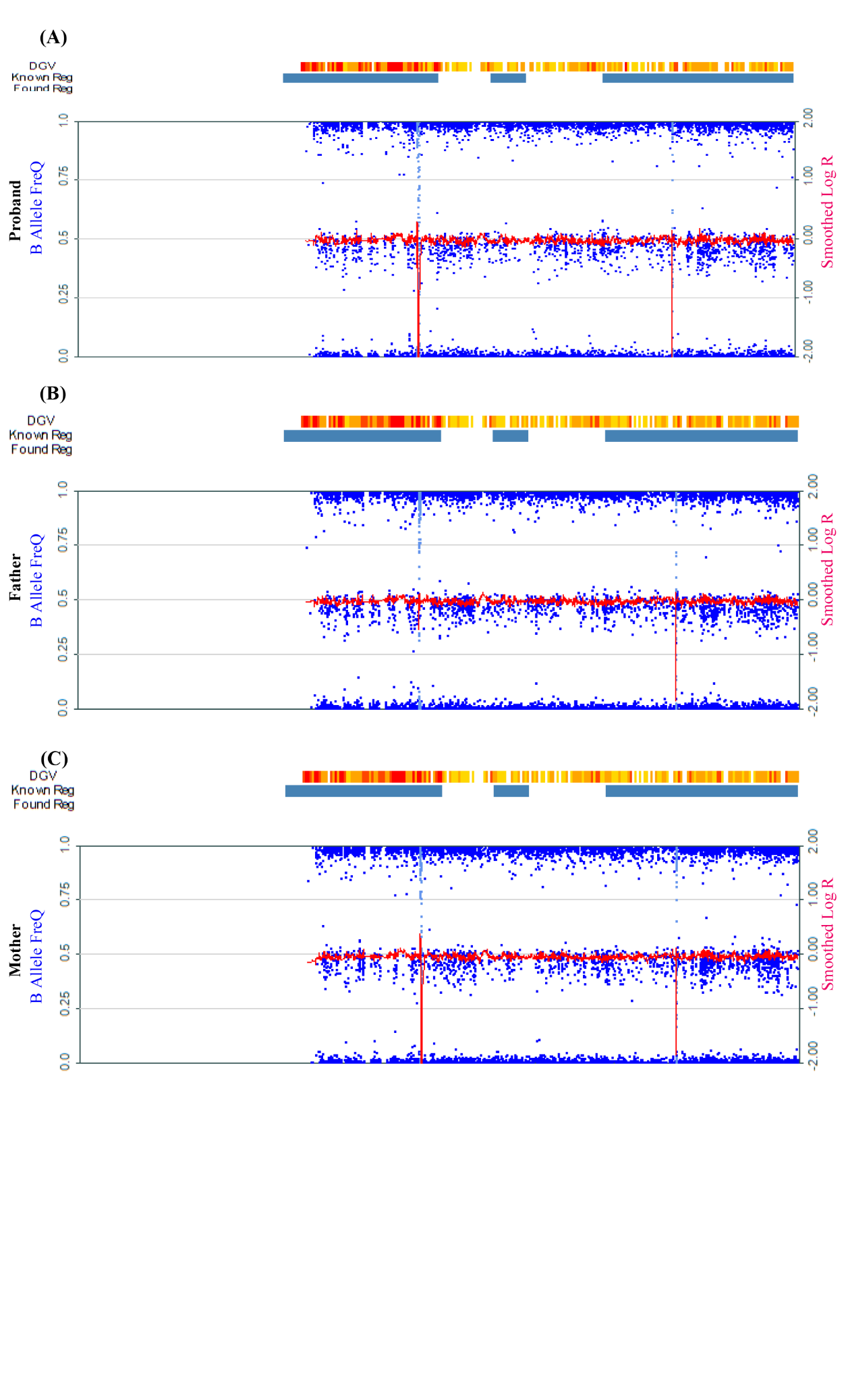


**Supplementary Figure 21.** No abnormalities were detected in chromosome 22.


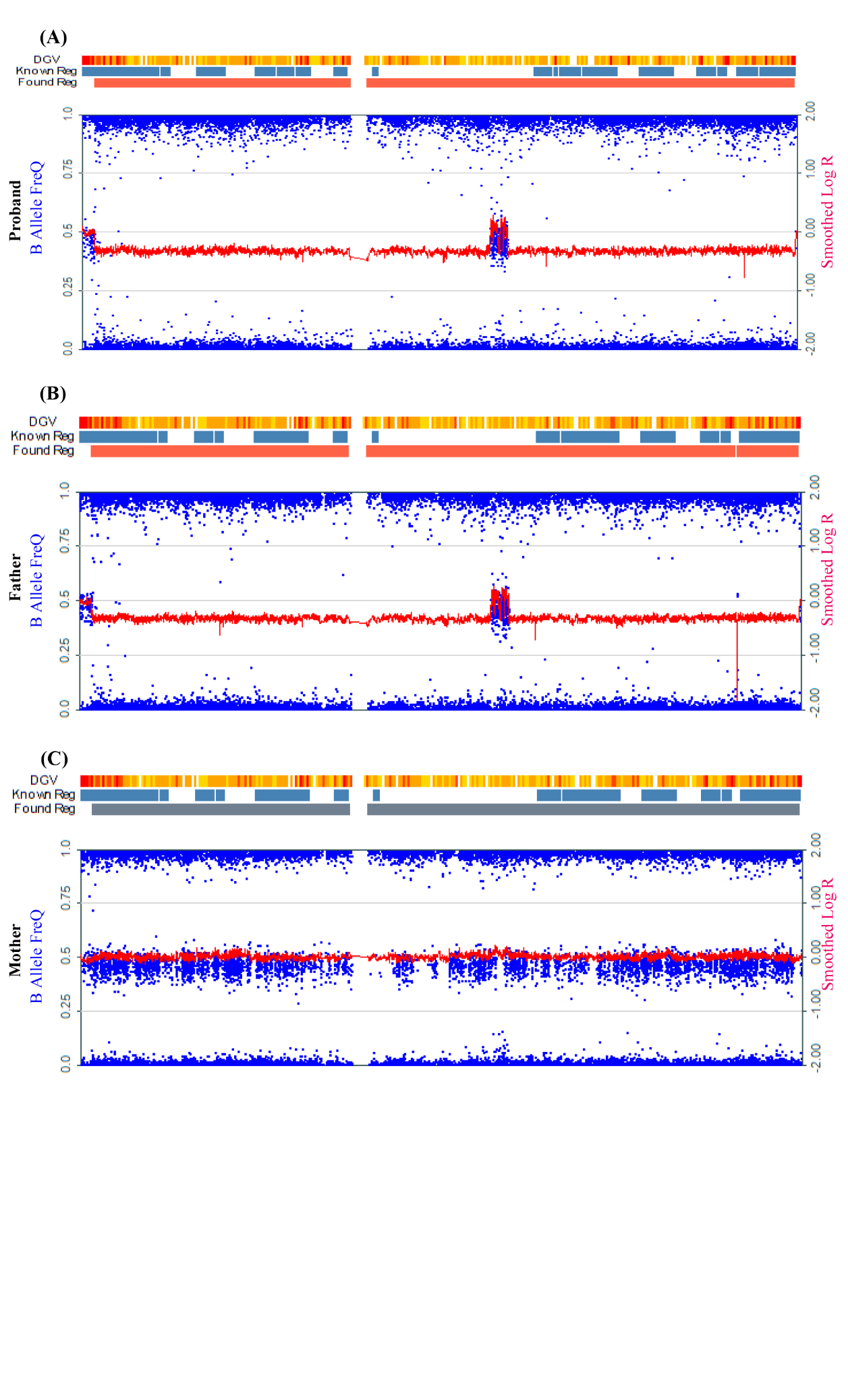


**Supplementary Figure 22.** No abnormalities were detected in chromosome X.


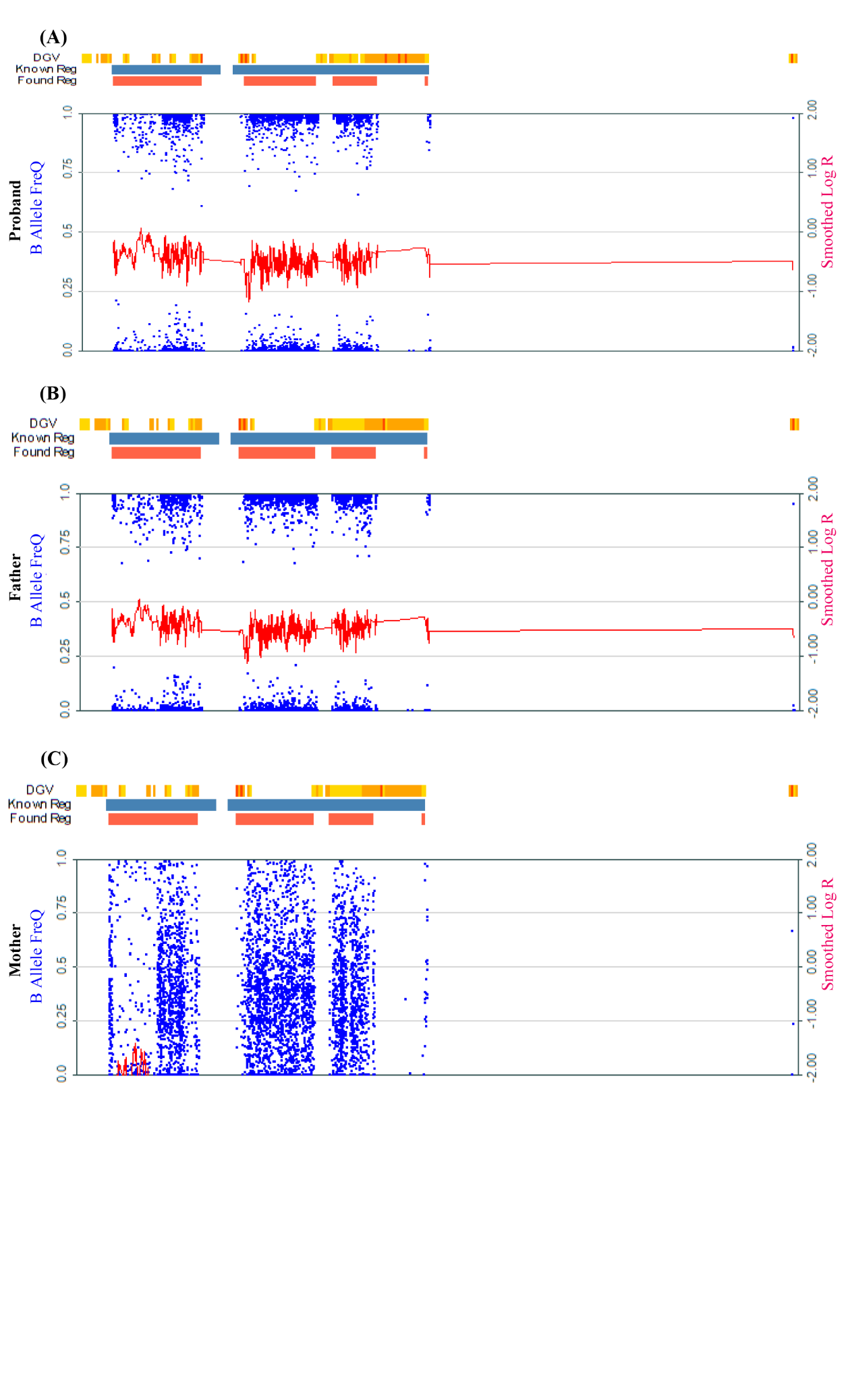


**Supplementary Figure 23.** No abnormalities were detected in chromosome Y.


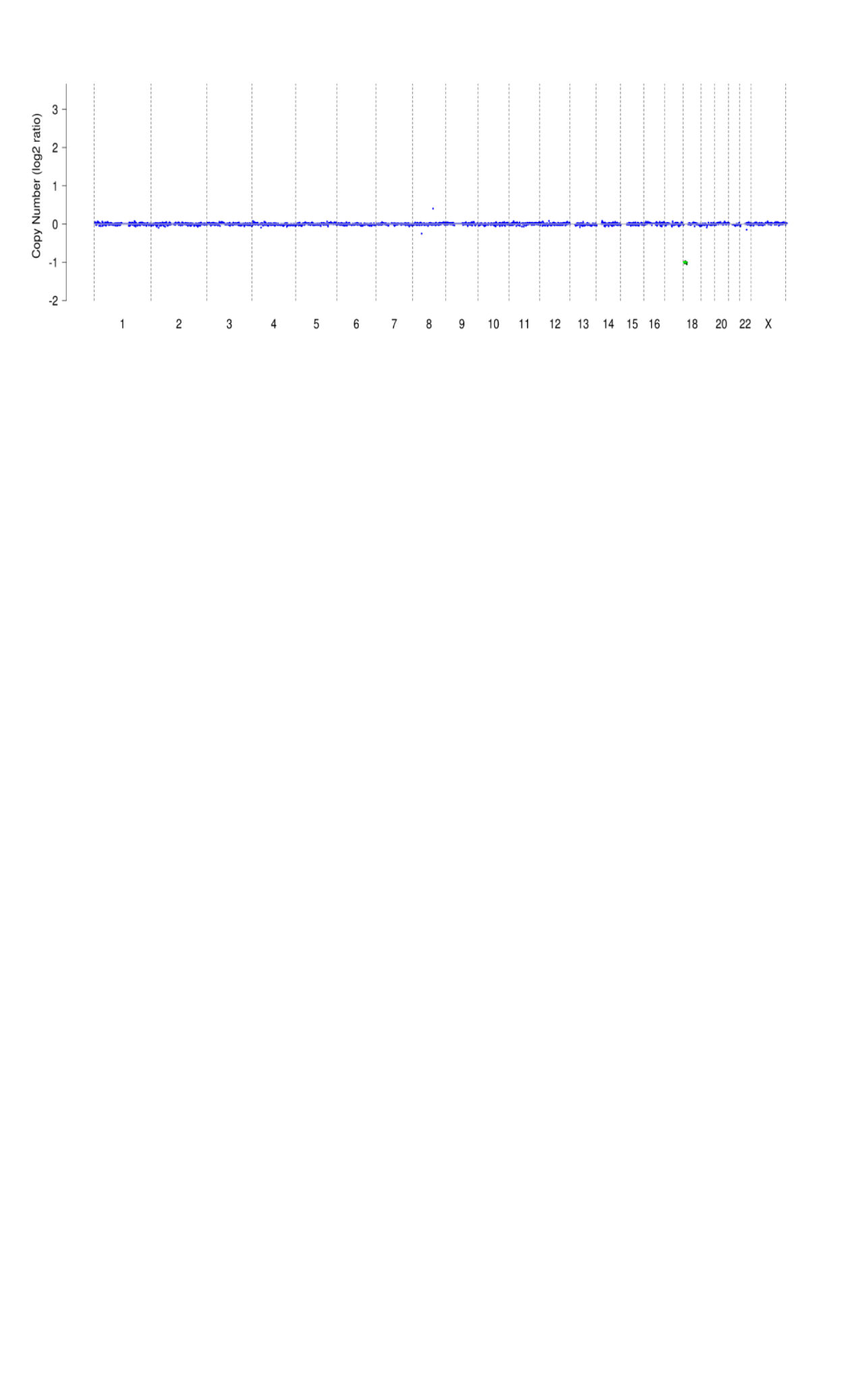


**Supplementary Figure 24.** The overview of the result of WGS of the proband.
